# Advancements in Musculoskeletal Tissue Engineering: The Role of Melt Electrowriting in 3D-Printed Scaffold Fabrication

**DOI:** 10.3390/jfb16050163

**Published:** 2025-05-07

**Authors:** Kunal Ranat, Hong Phan, Suhaib Ellythy, Mitchell Kenter, Adil Akkouch

**Affiliations:** 1 Western Michigan University Homer Stryker M.D. School of Medicine, Kalamazoo, MI 49008, USA; 2 Department of Surgical Services, Division of Medical Engineering, Western Michigan University Homer Stryker M.D. School of Medicine, Kalamazoo, MI 49008, USA; 3 Department of Surgical Services, Division of Orthopaedic Surgery, Western Michigan University Homer Stryker M.D. School of Medicine, Kalamazoo, MI 49008, USA

**Keywords:** melt electrowriting, 3D printing, scaffold, musculoskeletal regeneration

## Abstract

Musculoskeletal tissue injuries of the bone, cartilage, ligaments, tendons, and skeletal muscles are among the most common injuries experienced in medicine and become increasingly problematic in cases of significant tissue damage, such as nonunion bone defects and volumetric muscle loss. Current gold standard treatment options for musculoskeletal injuries, although effective, have limited capability to fully restore native tissue structure and function. To overcome this challenge, three-dimensional (3D) printing techniques have emerged as promising therapeutic options for tissue regeneration. Melt electrowriting (MEW), a recently developed advanced 3D printing technique, has gained significant traction in the field of tissue regeneration because of its ability to fabricate complex customizable scaffolds via high-precision microfiber deposition. The tailorability at microscale levels offered by MEW allows for enhanced recapitulation of the tissue microenvironment. Here, we survey the recent contributions of MEW in advancing musculoskeletal tissue engineering. More specifically, we briefly discuss the principles and technical aspects of MEW, provide an overview of current printers on the market, review in-depth the latest biomedical applications in musculoskeletal tissue regeneration, and, lastly, examine the limitations of MEW and offer future perspectives.

## 1. Introduction

In medicine, many diseases and pathologies result in the destruction of tissues and organs, ultimately impairing their overall function. While the current treatment options for these diseases and pathologies are beneficial in improving the conditions, many are still limited in their ability to completely restore tissue and organ function. One such group of diseases involves the musculoskeletal system. The system consists of different types of tissues including bones, muscles, cartilage, ligaments and tendons [1]. Given its composition, this system plays an integral role throughout life, providing protection and facilitating movement as a single multi-tissue unit. Injuries to these structures, whether soft tissues or cartilage, can often lead to degenerative diseases, such as osteoarthritis, and have an impact on life-long functional ability. For example, delayed repair of tendon to bone injuries can result in increased fatty degeneration and decreased biomechanical properties of the bone [2,3]. While less severe musculoskeletal diseases are often treated with physical rehabilitation and pharmaceuticals, severe defects often require surgical interventions using tissue grafting or orthopedic devices. Grafts often require autologous, cadaveric, or xenografting procedures. Nevertheless, challenges associated with each type include donor availability, immunogenic risks, and impaired regenerations [4]. Thus, development and progress in the fields of tissue engineering and regenerative medicine are paramount for advancing current therapeutic opportunities to regain functional tissue from a once damaged state [5].

There are multiple approaches to regenerate tissue with normal biological functions, but the use of scaffolds is of particular interest. Scaffolds have emerged as critical components in tissue engineering because they can guide tissue growth by mimicking the role of the extracellular matrix (ECM). Importantly, ECM is composed of a 3D fibrous framework that serves as a foundation to facilitate key cell behaviors that inevitably allow tissue to function [6]. For scaffolds to serve as substrates for tissue engineering, it is crucial that their builds incorporate fibrous architecture. Hence, a fibrous scaffold with suitable biochemical and physico-mechanical properties will support cell migration, adhesion, proliferation, and differentiation to recapitulate the native tissue.

Various techniques have been studied for the fabrication of 3D scaffolds for tissue engineering, including phase-separation, freeze-drying, self-assembly, and electrospinning [7]. Among the various methods, electrospinning is of special significance. This is a simple process that offers the ability to fabricate scaffolds composed of ultrathin fibers with diameters within the nanometer range using either polymer melts or polymer solutions [8]. The ability of these fibers to approach the morphological magnitude and resolution of native ECM-established electrospinning is a promising technique for tissue engineering. However, the working mechanism of electrospinning to produce nanofibers is innately restricted in its control of fiber deposition, making the technique mostly incompatible in fully regenerating tissue [9,10]. The rise of additive manufacturing (AM) techniques such as fused deposition modeling (FDM) has served as a potential solution to the limitations associated with electrospinning. The advantages associated with FDM include highly controllable porosities and dimensional accuracy, which enable the fabrication of scaffolds with geometries that can better replicate the complex 3D structural arrangements of tissue [11,12]. Unfortunately, although FDM resolves the restrictions associated with electrospinning, it is unable to maintain the advantage of producing fibers with nanoscale resolution. To overcome these limitations and preserve the advantages of both electrospinning and FDM, AM has led to the development of melt electrospinning writing, otherwise known as MEW. MEW is a novel method for fabricating reproducible scaffolds with a high level of tailorability through customized geometries, modification of design parameters, and the use of a wide array of polymers. This paper focuses on MEW applications as they relate to musculoskeletal tissues including bone, cartilage, ligaments, tendons, and muscle. We will explore the fundamentals of the recently developed AM technique, including the principles of the MEW process, current technologies available in the market, and detail the recent applications of MEW in tissue engineering with emphasis on the particular methods of MEW application in hard tissues. By surveying recent progress and advancements made in tissue engineering with MEW, we hope that this paper will serve as a resource for further interdisciplinary research in bioengineering and medicine.

## 2. Melt Electrowriting Technology

### Development and Principles of Melt Electrowriting

**Figure 1:**
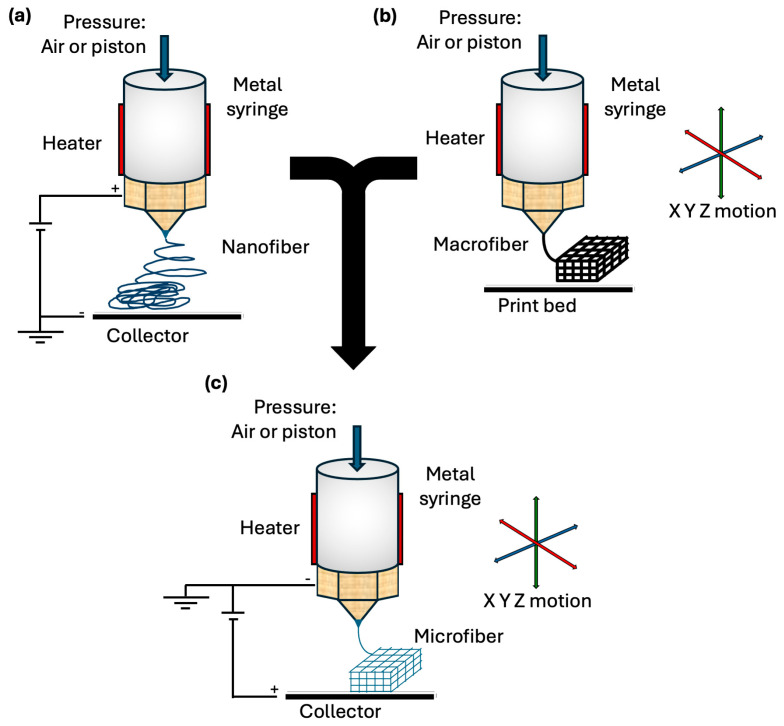
Melt electrowriting technology. (**a**) Schematic of melt electrospinning printing process. (**b**) Schematic of FDM printing process. (**c**) Schematic of MEW printing process, integrating favorable characteristics of both FDM and melt electrospinning allowing for controlled and organized deposition of microfibers.

Fibers fabricated at the micrometer and nanometer scales have become significant building blocks for structures with significant applications in regenerative medicine and tissue engineering [13]. Recently, technologies and techniques have been developed to refine the process of fiber production to produce a more stable and versatile product. As mentioned above, traditional electrospinning can be used to fabricate scaffolds through nanofibers assembly. The process of producing these nanofibers involves placing a polymer (liquid or molten) under a high electric field. The electrification of the polymer results in the generation of a jet that is ejected from a nozzle and undergoes stretching and thinning due to bending instabilities induced by the high electric field. While traveling, the polymer jet solidifies, which also contributes to the decrease in the diameter of the jet. Eventually, an ultrathin, fine-tuned fiber is deposited onto the charged collector (Figure 1a) [8,9,14]. As a result of this process, scaffolds fabricated via electrospinning are composed of nanofibers whose dimensions closely resemble those of the native ECM within the tissue microenvironment. However, a crucial drawback of electrospinning is that because the fibers are subject to natural electrical bending instabilities, they are deposited randomly onto the collector, thus lacking precise fiber alignment and limiting the ability to control and coordinate scaffold design [9,14]. The unpredictable, imprecise deposition of fibers leads to small pore sizes and dense fiber packing, which result in poor cell infiltration into the deeper layers of the scaffold [10]. Moreover, the most commonly used solvents in solution electrospinning are halogenated and highly toxic, making their evaporation and release during the process considerably a significant environmental and safety concern [15].

To achieve greater control over the fabrication process, FDM has been studied as a promising method for producing scaffolds with specific complex geometries. In FDM, a thermoplastic material is fed into a hot-melt printing nozzle that heats the polymer to a semiliquid molten state. Under the control of a programmed design, the nozzle follows the track indicated by computer inputs and extrudes the molten filament along this coordinated path. As the printing continues, a stable macromolecular scaffold is fabricated through layer-by-layer deposition of the molten material (Figure 1b). Most importantly, FDM enables the adjustment of printing parameters such as nozzle temperature, diameter, moving speed, and construction direction, inevitably affecting the properties of the final scaffold [16,17,18]. Despite the advantage that FDM governs the directionality and accuracy of the fabrication process, the technique is typically restricted to producing fibers with sizes between 200 and 500 µm, far from the nanometer scale, which is favorable for replicating tissue microenvironments [16].

Ultimately, given the advantages and disadvantages of electrospinning and FDM, recent advancements in AM have melded these two methods to develop MEW (Figure 1c). By promoting the positive aspects and refining the limitations of the above-mentioned technologies, MEW can precisely produce 3D scaffolds with fibers as small as a few micrometers in diameter, suitable for tissue regeneration [19,20,21].

**Figure 2:**
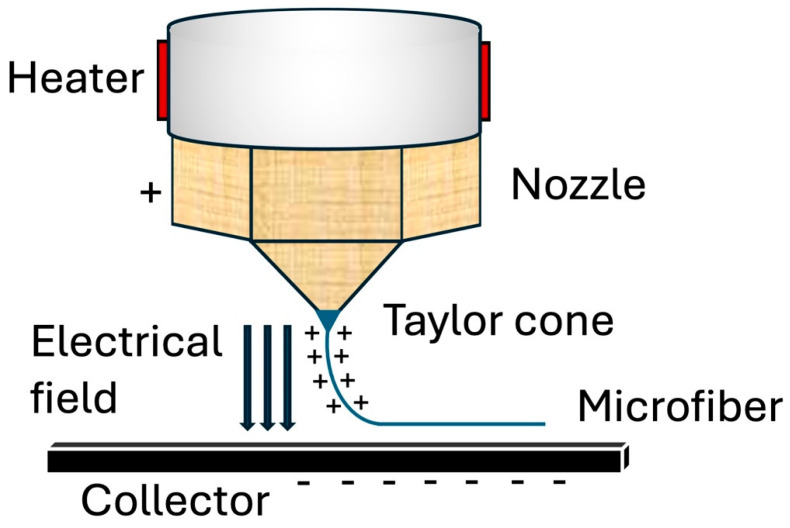
Melt electrowriting process. As the melt polymer is extruded, a Taylor cone forms in response to the surrounding electrical field. The charged jet is stretched and thinned as it continues to leave the nozzle until it eventually deposits and solidifies onto a collector, forming a microfiber.

In terms of principles, as Loewner et al. described in their review, the MEW process can be simplified into two steps: (1) air pressure and heat are applied to a dispensing piston to extrude a molten thermoplastic polymer material, and (2) a small droplet is created at the end of the piston nozzle [22]. Similar to traditional electrospinning, MEW also utilizes an electric field that develops from a potential difference created by applying a voltage to both the print nozzle and collecting print bed. As the polymer melts, the pneumatic delivery system extrudes the molten polymer through a print nozzle. The extruded polymer begins to interact with the electrical field created by the applied voltage, eventually forming a Taylor cone [23]. As Taylor cones are subject to a multitude of forces, continued extrusion of the molten polymer is required to add to the gravitational force to overcome surface tension. Once the polymer overcomes the surface tension, the gravitational force from the weight of the polymer and the force of the electrical field generates an initial droplet and polymer fiber that falls to the collector plate. Here, electrowriting is initiated once the polymer makes contact with the print bed, and the cartridges of the MEW printer move to conduct the fabrication (Figure 2) [24]. A critical component of this process is to maintain the collector speed at a certain threshold. This threshold, termed the critical translation speed (CTS), is the speed at which the collector matches the jet, which is conducive to producing linear fibers [20]. The print continues via a layer-by-layer process, where fibers are deposited and accurately stacked on top of the previous layer until the desired design is achieved.

In contrast to traditional electrospinning, where fibers are subject to electrical instabilities, MEW uses an applied voltage and lower flow rates to stabilize and allow the molten polymer to deposit as a continuous stream of fibers without breaking. The lower flow rates observed in MEW are typically dependent on the polymers used. In MEW, polymers with high viscosity and low conductivity are commonly used as they are conducive to achieving sufficient control and stability of the extruded jet to enable proper fiber layering [24]. Additionally, polymers used in melt-extrusion-based techniques such as MEW, are free of solvents and formulations that make previous techniques such as solution electrospinning and FDM toxic. Hence, MEW offers a safer and more environment-friendly process for the fabrication of scaffolds for tissue engineering. Furthermore, the distance between the MEW printhead nozzle and the collector is smaller than the distance between the spinneret and the collector observed during electrospinning to ensure proper stretching and solidification of the jet after ejection. In MEW, the molten polymer solidifies as it cools almost immediately after extrusion; therefore, a smaller nozzle-collector distance is sufficient for fiber printing [14,20].

## 3. Printing Parameters

Fabrication of scaffolds using MEW technology necessitates ensuring that the polymer used, and the resulting constructs, possess distinctive and advantageous properties suited perfectly to a specific biomedical application or particular use. These properties are largely based on the polymer and instrument-based printing parameters used for scaffold fabrication. Furthermore, different polymers require adjustment of various printing parameters to produce the desired fiber characteristics and, consequently, the desired scaffold morphology, architecture, and mechanical properties [25]. Many material options are available for use in MEW, with Poly(ε-caprolactone) (PCL) being the current gold standard and the most common polymer used. For further details on MEW polymers, Kade et al. conducted a thorough review of PCL and other polymers, and highlighted their advantages [24].

The primary printing parameters—applied voltage, pressure, collecting speed, and temperature—significantly influence the morphology of the extruded fibers. Regarding the fiber diameter, for PCL specifically, previous studies suggest that increasing melt temperatures and applied pressure but decreasing collector speed yields greater fiber diameters [19,25,26,27,28,29]. Changing these parameters results in reduced viscosity and greater extrusion of the material, thus allowing greater mass flow while limiting stretching of the polymer jet, which explains the increase in fiber diameter. The effect of the voltage on the fiber diameter appears to be more complicated, as there is conflicting evidence. Two studies by Wunner et al. provided supportive evidence that an increase in the applied voltage results in increased fiber diameter [27,30]. However, contrary evidence also exists, suggesting that increasing fiber diameters are produced with decreasing voltages [29,31]. It is also important to note that the effects of these instrument parameters on fiber diameter may change depending on the polymer, as shown by Hochleitner et al., who used a thermoplastic elastomer rather than PCL [29]. In terms of the fiber shape, the MEW printing process typically results in the deposition of either linear or coiled fibers, with a linear shape preferred for accurate scaffold fabrication. Coiling occurs because of the extruded fiber buckling under axial compression, as it impacts the collector surface and can be prevented by modifying certain printing parameters. Specifically, the relationship between the collector speed and the CTS is a primary driver of the fiber shape. Maintaining a collector speed that is equal to or greater than the CTS is most conducive for the deposition of straight fibers, whereas deviating below the CTS results in increased coiling as the collector speed increases [19,31,32].

Along with the fiber properties, studies have also examined the impact of instrument-based parameters on whole scaffolds. Mechanical testing of scaffolds revealed that the apparent tensile modulus generally decreased when the collecting speed and melt temperature increased [28]. Pairing and altering collector speed and pressure have enabled the production of multimodal scaffolds with opportunities to vary properties, such as pore size or fiber diameter, throughout a single construct [26]. Finally, fabrication with lower starting applied voltages yielded thicker scaffolds with less distortion and greater accuracy of layer deposition [25].

The relationship between the printing parameters and scaffold architecture highlights the significance of tailoring these parameters for the fabrication of structures with desired features and properties. It is also worth mentioning that these parameters are not mutually exclusive and that there is a dynamic interplay between the parameters that contribute to the final product. Manipulating multiple parameters and optimizing these settings before and during fabrication may be required to balance their effects and produce fibers and scaffolds with the desired morphologies (Table 1). For example, when we used PCL for MEW, increasing the processing temperature reduced the polymer’s viscosity, enabling the formation of thinner and more uniform fibers, whereas lower temperatures resulted in thicker and less consistent structures. Similarly, increasing the printing speed led to finer fibers due to shorter deposition time per unit area, while slower speeds caused fiber accumulation and reduced structural precision. A larger collector distance allowed more time for fiber stretching and solidification, which improved alignment but sometimes at the cost of resolution. Higher air pressure and applied voltage contributed to better fiber elongation and control; however, excessive values could destabilize the jet and negatively affect scaffold uniformity.

## 4. Current MEW Technology on the Market

**Figure 3:**
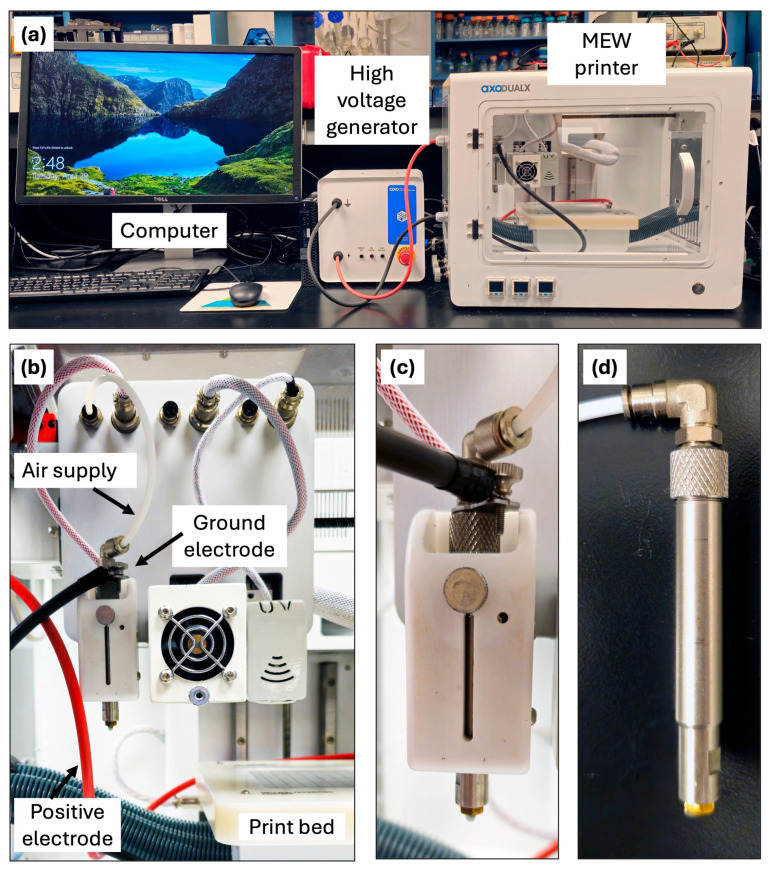
MEW bioprinter unit. (**a**) Image of a Axo-A3 3D Bioprinter system with MEW capabilities. (**b**) multiple printhead options such as low temperature, UV curing, and MEW printhead; Print bed with electrodes connection. (**c**) MEW printhead assembly and air supply connection. (**d**) Stainless-steel cartridge and nozzle used for MEW printing.

Because MEW may generally be categorized under the umbrella of 3D printing, the specifications and necessary technology for MEW are not drastically different from those of many additive manufacturing 3D printers available on the market today. Nevertheless, the opportunities, advantages, and complexities of MEW compel the need for a research-grade stand-alone MEW printer. These printers are available commercially or may be custom-built, with the latter being more commonly used in the MEW literature. In terms of primary components, a MEW 3D printer typically contains a 3-axis motion system, an air pressure vessel, a printhead with heating and voltage application capabilities containing a metal syringe, a print collector plate that also serves as part of the applied voltage system, and a high-voltage generator (Figure 3).

**Figure 4:**
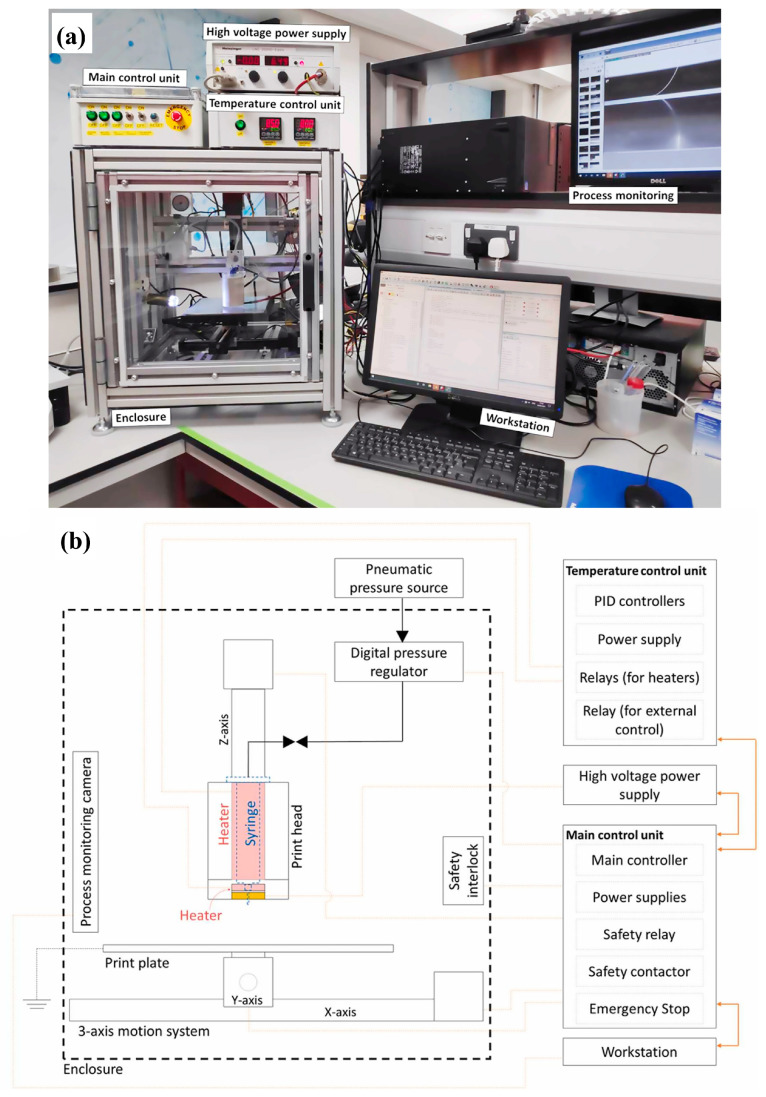
Homemade MEW system components. (**a**) Photograph of completed custom-built MEW printing setup. (**b**) Schematic diagram demonstrating main components of MEW printer. Adapted from Ref. [33].

Custom-built printers have the advantage of enabling researchers to tailor the system for specialized MEW processes with freedom for further configuration based on research needs. For more information, Eichholz et al. outlined the design, development, and construction of a custom MEW printer and demonstrated its capabilities (Figure 4) [33]. As for commercial printers available in the market, companies and their devices that have MEW capabilities offer an array of specifications to meet scaffold printing needs (Table 2).

## 5. Musculoskeletal Applications

### 5.1. Bone Regeneration

Bone is a sophisticated, hierarchically organized organ that forms a structural framework and predicates movement while also contributing to calcium and phosphate regulation within the body [34]. Although typically perceived as a hard tissue organ, bone constantly undergoes a process of dynamic turnover, termed bone remodeling, to repair and replace old or damaged bone. The physiology of bone remodeling was further reviewed by Siddiqui and Partridge [35]. Bone defects are characterized as damaged or missing bones that are often caused by external factors, such as fractures, trauma, and infections, or intrinsic factors, such as congenital malformations or disease processes. Although natural bone regeneration effectively heals most bone defects, in cases where the defect is too large for self-healing alone to restore the bone to its original state, an external scaffolding implant is required [36]. This scaffold will facilitate host cell proliferation and differentiation processes. The current “gold standard” treatment intervention to enhance bone healing is harvesting and transplantation of autogenous bone. However, this is associated with its own limitations, such as high morbidity during harvest, and despite having similar osteogenic and osteoinductive properties, autografts lack the osteoconductive properties and biomechanical stability necessary to promote adequate bone regeneration [37]. Other available therapeutic options include allogenic bone grafts and specific surgical strategies depending on the nature of the defect. However, these highly specialized and individualized treatments are expensive, time-consuming, and burdensome for the patient [37]. Given the complexity of poorly healing bone defects and the limitations of current clinical treatment methods, a novel strategy to enhance bone regeneration is now becoming a necessity. The recent development of manufacturing technologies for tissue engineering and regeneration has shown great potential for bone regeneration, and MEW is an emerging technology that shows promise for fulfilling this need [38,39,40]. Here, we discuss recent efforts to utilize MEW for bone regeneration and healing.

### 5.2. Effect of MEW Scaffold Architecture on Osteogenesis

One particularly important advantage of MEW is its tailorability. This technology allows for customization and control of specific scaffold characteristics that constitute the overall architecture of the scaffold [19,41,42]. In turn, MEW is capable of fabricating complex, anatomically relevant scaffolds that more closely mimic the microarchitecture of bone tissue. Thus, it is worthwhile to investigate the relationship between the various scaffold architectures and their influence on bone regeneration and osteogenesis.

**Figure 5:**
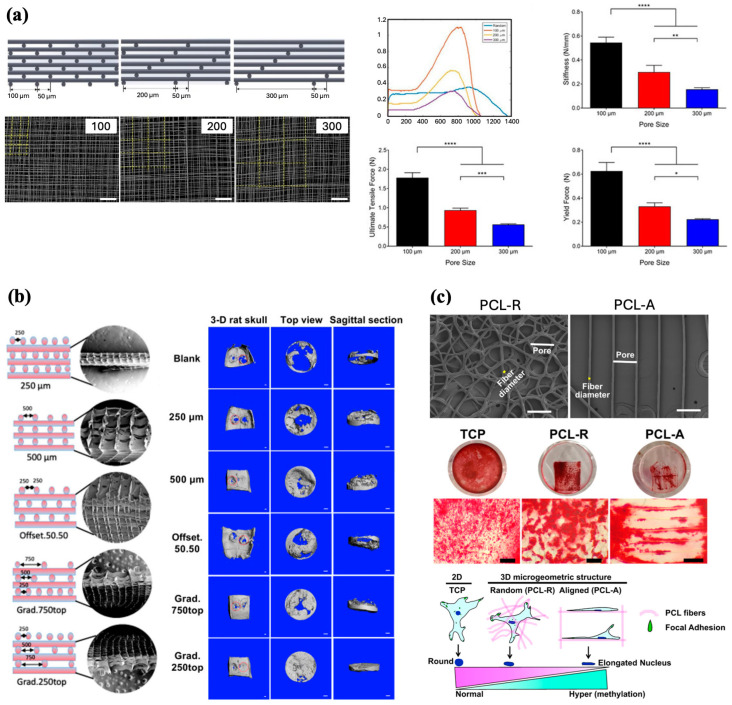
Effect of pore size and fiber alignment on scaffolds properties and cell behavior. (**a**) CAD models and SEM images of 100, 200, and 300 µm scaffold designs and their respective mechanical properties (Scale bar 200 μm, * *p* < 0.05, ** *p* < 0.01, *** *p* < 0.001, and **** *p* < 0.0001). Reproduced with permission from Ref. [43]. Copyright 2019 IOP. (**b**) Diagrammatic representation and SEM images of different scaffold structures, and degree of bone repair observed in rat calvarial defects 8 weeks after implantation (Scale bar 1 mm). Adapted from Ref. [48]. (**c**) SEM images of scaffolds with random and aligned fibers, and Alizarin Red staining of human osteoblasts (hOBs) demonstrating osteogenic differentiation after 3 weeks. Illustration of nuclei shape changes in response to fiber alignment (Scale bar 50 μm). Adapted from Ref. [49].

Pore size, or the distance between fibers within a scaffold, is an interesting modifiable component of scaffolds that may influence cell behavior. Brennan et al. examined pore size and its effect on the differentiation of human bone marrow stem cells (BMSCs) to an osteogenic lineage (Figure 5a). PCL scaffolds with square pore sizes of 100, 200, and 300 µm were fabricated and seeded with BMSCs. After 21 days of culture, scaffolds with 200 µm pore sizes promoted the highest proliferation of BMSCs. However, the 100 µm scaffolds proved to be the most effective overall, as they exhibited the highest cell seeding efficiency and mineralization while maintaining cell morphology with the greatest osteogenic capacity, and demonstrated the best mechanical properties, with a significantly greater stiffness and tensile force compared to the other scaffolds [43]. A comparable evaluation of pore size and cell behavior, as studied by Han et al., yielded similar results. PCL scaffolds fabricated with pore sizes of 50, 100, 200, 300, and 400 µm were individually assessed for their effects on BMSCs adhesion, proliferation, and differentiation. Similar to Brennan et al., a pore size of 200 µm was found to be optimal for the greatest BMSCs adhesion and proliferation activity [44]. However, the differentiation of BMSCs towards an osteogenic lineage has not been particularly examined; therefore, it is not possible to draw a conclusion regarding whether a pore size of 200 µm is the most suitable for osteogenesis and bone regeneration. Eichholz et al. investigated the influence of pore size and compared the microarchitectures of PCL scaffolds printed by FDM versus MEW, and their ability to heal critically sized femoral defects in vivo. While maintaining comparable surface areas, the fabrication of scaffolds via FDM generated larger fiber diameters and lower porosities, whereas MEW scaffolds demonstrated smaller fiber diameters with greater porosities. MEW scaffolds were not only found to induce the greatest levels of total bone formation in vivo with the new bone conveying a more rounded healing front, but also promoted a greater distribution of vessels with smaller diameters, which the authors hypothesize is more conducive for bone healing in segmental defects [45].

Along with varying pore sizes among scaffolds, MEW is also capable of fabricating gradient constructs with multiple different pore sizes in one scaffold, as well as printing varying lay-down patterns to generate a scaffold with different strut offsets. As such, Abbasi et al. conducted a group of studies assessing the impact of different offset and gradient parameters on the physical and biological properties of scaffolds [46], osteoblast differentiation [47], and in vivo bone regeneration [48]. The authors designed and fabricated distinct PCL scaffolds with three homogenous pore sizes (250, 500, and 750 µm), two fiber offsets (30/70% and 50/50%), and one three-layered structure with gradient pore sizes of 250, 500, and 750 µm (Figure 5b). All scaffold groups were modified with a calcium phosphate (CaP) coating to enhance scaffold hydrophilicity and bioactivity. Mechanical assessment of the scaffolds indicated that a pore size of 250 µm yielded the highest tensile strength, 500 µm showed the highest compressive modulus, and the gradient scaffold demonstrated the greatest compression stress. The adhesion of human osteoblasts to scaffolds was also evaluated, revealing that the 250 µm pore size, 30/70 offset, 50/50 offset, and gradient scaffold groups showed the highest seeding efficacies. Analysis of cell culture after 30 days indicated that the gradient architecture had the greatest degree of osteoblast proliferation and infiltration [46]. In a subsequent study, the five aforementioned CaP-coated PCL scaffold groups were used to examine their effects on osteoblast osteogenic potential. The results showed that while both the gradient and offset scaffolds supported ECM deposition, the gradient scaffolds induced the greatest amount of alkaline phosphatase (ALP) activity in osteoblasts. However, the 50% offset scaffolds facilitated the most ECM mineralization and expression of osteocalcin and osteopontin, which are late osteogenic markers indicative of mineralization and osteoblast differentiation. Overall these data suggest that offset and gradient architectures are the most promising characteristics for promoting bone differentiation [47]. Lastly, Abbasi et al. fabricated five differently designed CaP-coated PCL scaffolds with the following architectures: 250 µm, 500 µm, 500 µm with 50% offset, gradient with 250(top)-500-750 µm, and a second gradient with 750(top)-500-250 µm for implantation into rat calvarial defects to assess the most suitable scaffold design for stimulating osteogenesis in vivo. Scaffolds with the 250(top)-500-750 µm architecture demonstrated the highest degree of new bone formation, significantly higher bone volume, and complete closure of pores with compact bone compared to other scaffolds [48]. Given these results, it appears that the MEW PCL scaffold with a porosity gradient, which may be the most mimetic of native bone tissue, has the greatest capacity to induce the highest bone regeneration in vivo.

Apart from pore size and fiber offset, MEW technology further enables tailoring of fiber deposition behavior, which may have implications on mechanosensing and mechanotransduction of cells for osteogenesis. Han et al. utilized the advantage of precise fiber placement afforded by MEW to develop PCL scaffolds with either random or aligned fibers to investigate the epigenetic response of human osteoblasts to a specific fiber orientation [49] (Figure 5c). Compared to a two-dimensional tissue culture plate control and scaffolds with random fibers, PCL scaffolds with an aligned fiber orientation influenced human osteoblasts and nuclei to take on an elongated shape and promoted increased global deoxyribonucleic acid (DNA) hypermethylation, which has been shown to be linked to osteogenic differentiation [50,51]. Accordingly, the aligned fiber group demonstrated significantly increased gene expression of multiple osteogenic markers compared to the other two groups. However, when the human osteoblast-loaded scaffolds were allowed to undergo osteogenic induction, a significant difference in osteogenic differentiation and calcium deposits was not found between groups. Nevertheless, these findings present a novel concept that fiber alignment may induce epigenetic modifications to influence cell mechanosensing in order to sway osteoblasts potential for osteoinduction and differentiation [49].

### 5.3. Supplementing MEW Scaffolds for Bone Regeneration

MEW scaffolds fabricated with polymers such as PCL, poly(lactic acid) (PLA), and poly(ethylene glycol) (PEG) are appreciated for their biocompatibility, slow degradation rate, and mechanical strength. However, common polymers in MEW often exhibit poor hydrophilicity, bioactivity, and bone-forming properties, suggesting that they may be unconvincing candidates for bone regeneration. Yet, several studies have shown that post-printing surface modification and functionalization of MEW scaffolds, as well as fabrication of composite materials, can overcome these limitations.

Meng et al. fabricated scaffolds using poly(L-lactic acid) (PLLA), which, similar to PCL, exhibited poor cell affinity and bioactivity. To improve these properties, the MEW-printed PLLA scaffolds underwent surface modification using an alkaline treatment method via immersion in sodium hydroxide solution and subsequent seeding with an osteoblastic cell line. The alkaline treatment method allows scaffolds to maintain their mechanical properties while improving scaffold surface roughness and osteoinductivity, which are conducive for immature bone formation. Analysis of cell-loaded modified scaffolds revealed coverage of the scaffolds with formed bone tissue and ECM, with the total scaffold thickness being three times greater than that of untreated scaffolds, suggesting significantly increased bioactivity of the treated scaffolds for bone growth [52]. Su et al. supplemented MEW scaffolds with the aim of enhancing bone regeneration as well by applying the piezoelectric properties of bone to scaffolds through a coating treatment with piezoelectric nanoparticles [53]. MEW scaffolds were fabricated using PCL and coated with either zinc oxide (ZnO) nanoparticles (PCL/ZP) or zinc oxide nanoflakes (PCL/ZF). Both the PCL/ZP- and PCL/ZF-coated scaffold groups were found to have significantly higher levels of ALP expression and calcium mineralization of osteoblasts compared to PCL-only control groups, implying improved osteogenic properties. Moreover, scaffolds modified with zinc oxide nanoflakes (ZF) showed enhanced ALP activity and calcium mineralization that were both two times greater than PCL/ZP scaffolds, likely attributed to the biomimetic topography of PCL/ZF scaffolds more closely resembling bone [53]. Eichholz et al. obtained similar results with their novel surface modification method for treating MEW PCL scaffolds with a nano-needle hydroxyapatite (nnHA) coating inspired by the nano-architecture and composition of native bones [54]. Analysis of nnHA scaffolds loaded with human BMSCs showed enhanced cell osteogenesis as indicated by increased cell proliferation, total ALP activity, and calcium deposition compared to control groups. Additionally, in a bone morphogenic protein 2 (BMP-2) adsorption study, nnHA coated scaffolds were found to facilitate the sustained delivery of stable BMP-2, which further promoted the osteogenic activity of these cells. Given these findings, it is worth noting that Su et al. compared their PCL/ZP and PCL/ZF scaffolds to hydroxyapatite (HA)-coated scaffolds, with both zinc oxide surface modification methods exhibiting superior osteogenic properties over HA treatment [53]. Overall, surface treatment methods have been shown to be an effective strategy for promoting the bioactivity of MEW scaffolds to improve new bone formation. Ren at al. proposed developing a composite biomaterial by coating MEW PCL scaffolds with a pro-angiogenic fibrin/alginate matrix (PCL/FA) as a means of accelerating bone repair via enhanced angiogenesis [55]. In vitro, the PCL/FA scaffolds supported pre-osteoblast attachment and their metabolic activity. An ex ovo chorioallantoic membrane assay of the composite scaffolds showed a higher number of blood vessels and bifurcation points, compared to the PCL-only controls. The PCL/FA scaffolds were subsequently implanted within a rat cranial defect model to further elucidate their osteogenic capacity in vivo. Micro-computed tomography (Micro-CT) analysis demonstrated a significant increase in overall bone regeneration in defects treated with PCL/FA scaffolds, and histological observations confirmed the enhanced formation of mature bone matrix and vasculature within the defects [55].

While post-printing surface modification is one approach for enhancing the bioactivity of MEW scaffolds, other approaches involve developing composite biomaterials by incorporating bioactive molecules that are compatible with MEW printing. In two separate studies, MEW composite structures were fabricated using PCL incorporated with either hydroxyapatite nanocrystals (PCL/HA) [56] or chitosan [57] and loaded with hOBs or hBMSCs, respectively. Both materials were chosen based on their previously demonstrated biological affinity for bone tissue (HA nanocrystals) [58,59] and cell growth potential (chitosan) [60]. Both studies shared common findings, with the integration of these bioactive materials into PCL scaffolds significantly improving cell activity, as evidenced by increased cell proliferation and infiltration of scaffolds. Furthermore, the incorporation of HA nanocrystals within PCL scaffolds showed enhanced cell attachment capacity, leading to greater hOB viability and bridging behavior, which are important characteristics for regenerating bone tissues. Abdal-hay et al. also studied the degradation behavior of PCL/HA scaffolds in an alkaline environment with the premise that naturally healing wounds tend to shift towards more alkaline conditions [56]. Notably, compared to scaffolds printed with PCL alone, the incorporation of HA nanocrystals resulted in a much greater scaffold mass loss and rate of degradation. In the context of bone regeneration, this property becomes increasingly important over time, as new bone continues to form and grow, invading the space of the scaffold to reconstruct the tissue completely.

On a separate note, a few studies have investigated supplementing MEW as an indirect approach towards bone regeneration, focusing on reproducing the developmental tissues that lead to bone formation. In an attempt to utilize endochondral ossification for bone formation, Hall et al. developed a biohybrid sheet for inducing bone formation in vivo by seeding MEW PCL scaffolds with osteogenic spheroids [61]. Human periosteum-derived cells were used to generate cartilaginous spheroids that were then captured by MEW PCL scaffolds for implantation within a critically sized mouse tibia defect. The inclusion of spheroids within PCL scaffolds led to notably greater bone formation and defect bridging compared with empty defects. Although a large variation in the ability of these biohybrid sheets to successfully regenerate bone tissue was observed, the potential of this composite scaffold to heal large bone defects via endochondral ossification warrants further investigation to improve this modality.

Conversely, given the osteoregenerative properties of native periosteum, targeting its regeneration may aid in promoting indirect regeneration of bone. Baldwin et al. developed a composite scaffold that incorporated star-PEG heparin hydrogels seeded with human umbilical vein endothelial cells (HUVECs) and BMSC-loaded tubular MEW PCL scaffolds to replicate the multiphasic morphology of native periosteum [62]. Similar to cells of the osteoprogenitor niche within the inner periosteum, BMSCs in the multiphasic scaffolds remained undifferentiated, whereas the HUVECs began to form nascent capillary-like networks in vitro. Implantation of the construct in a novel orthotopic xenograft mouse model further validated the scaffold’s potential as a tissue-engineered periosteal graft, as the HUVECs were able to connect to the host vasculature and mature into functional vessels, successfully recapitulating the vascular niche of the native periosteum as well. Given the novelty of this approach, future studies may use this composite to examine its potential for inducing bone regeneration both in vitro and in vivo.

### 5.4. Combining MEW with Other 3D Printing Techniques

While supplementing MEW scaffolds with bioactive materials may help improve their osteoregenerative potential, more must be done to overcome the inherent limitations of scaffolds to further advance bone tissue engineering. Recent studies have demonstrated the potential of integrating other 3D printing techniques with MEW to produce scaffolds with improved mechanical properties as well as bioactivity without post-fabrication treatments or modifications. Kilian et al. developed an automated one-step fabrication process that simultaneously combined 3D plotting of calcium phosphate cement (CPC) and MEW to generate PCL + CPC construct. The incorporation of MEW PCL fibers with CPC demonstrated a stable interconnected network that improved the integrity of the construct compared with the CPC-only scaffolds. In a similar comparison of both scaffold groups loaded with hMSCs and murine pre-osteoblasts, the addition of PCL fibers to CPC displayed improved hMSC bridging behavior and pore coverage as well as significantly increased murine pre-osteoblasts proliferation [63].

In a separate study, investigators proposed combining the advantages of MEW and solution electrospinning to develop composite scaffolds that promote bone regeneration. Ordered PCL microfibers and random gelatin nanofibers were fabricated via MEW and solution electrospinning, respectively, and alternately stacked to create micro/nanohybrid scaffolds. The incorporation of gelatin nanofibers with PCL microfibers led to improvements in scaffold tensile strength and modulus compared to PCL-only scaffolds, possibly due to the nanofibers allowing for greater dispersion of the force applied. Culturing the composite scaffolds with Saos-2 osteoblast cell line subsequently demonstrated enhanced cell seeding efficiency with gelatin nanofibers providing a supplemental surface for cell attachment and adhesion. In terms of osteogenic potential, the ALP activity assay and alizarin red staining (ARS) indicated that the combined PCL/gelatin scaffolds exhibited a synergistic effect in promoting osteogenic capacity compared to MEW scaffolds alone. Finally, to assess osteoblast penetration, 3D multi-layer scaffolds were fabricated by repeated stacking of alternating MEW PCL microfibers and gelatin nanofibers. As a result, while osteoblasts demonstrated proficient adherence and high orientation in both composite and control scaffold groups, only the PCL/gelatin scaffolds allowed cells to penetrate through the nanofiber pores and grow between fibers, eventually proliferating evenly throughout the whole composite. To that end, as native bone microscopically exhibits a micro/nano heterogeneous structure, integrating solution electrospinning nanofibers with MEW microfibers shows promise in recapitulating this environment [64].

Using a similar concept, Wang et al. developed a cross-scaled scaffold by combining MEW and FDM techniques [65]. They prepared a PCL/beta-tricalcium phosphate (PCL/β-TCP) composite polymer, and FDM was used to print thick fibers and the subsequent scaffold pores. Compared to PCL/β-TCP scaffolds printed solely by FDM, scaffolds fabricated via the dual printing process showed elevated osteogenic performance and differentiation of BMSCs, with a significant ALP activity and calcium deposition within the pores of the cross-scale scaffold. Eichholz et al. also combined FDM and MEW to construct a hybrid scaffold to enhance the healing of large bone defects. The hybrid composite was composed of an outer shell that was printed with larger, stiffer fibers of FDM for mechanical support and an inner core fabricated with MEW microfibers to facilitate osteogenesis and guide the bone-healing process. Implantation of hybrid scaffolds within rat femoral bone defects demonstrated nearly complete defect bridging, greater bone volume, and increased vascularity compared with MEW-only scaffolds. While the inner MEW core was essential for promoting osteogenesis, the outer FDM shell was crucial in preventing the encroachment of muscle and soft tissue into the defect, which would otherwise have deterred adequate bone regeneration [66]. Given the positive results from these studies, scaffolds created through the integration of FDM and MEW were filled with high-precision, fine fibers deposited by MEW. Culturing of the cross-scale scaffolds with BMSCs revealed enhanced cell proliferation and scaffold biocompatibility. Additionally, the inclusion of MEW fibers allowed cells to bridge across and fill pores, showing promise for efficient and effective bone defect repair.

### 5.5. Replicating the Bone Environment via MEW to Study Pathophysiology

From a clinical perspective, understanding the physiological and pathological processes of diseases that affect bones is becoming increasingly important for developing treatments. Current models for elucidating the pathophysiology of disease processes either lack the complexity to simulate the native bone microenvironment [67] or have limited availability for research [68,69]. As such, fabricating readily available 3D models capable of effectively mimicking the microscopic bone environment fulfills a critical desire, allowing for the study of biological processes in greater detail.

**Figure 6:**
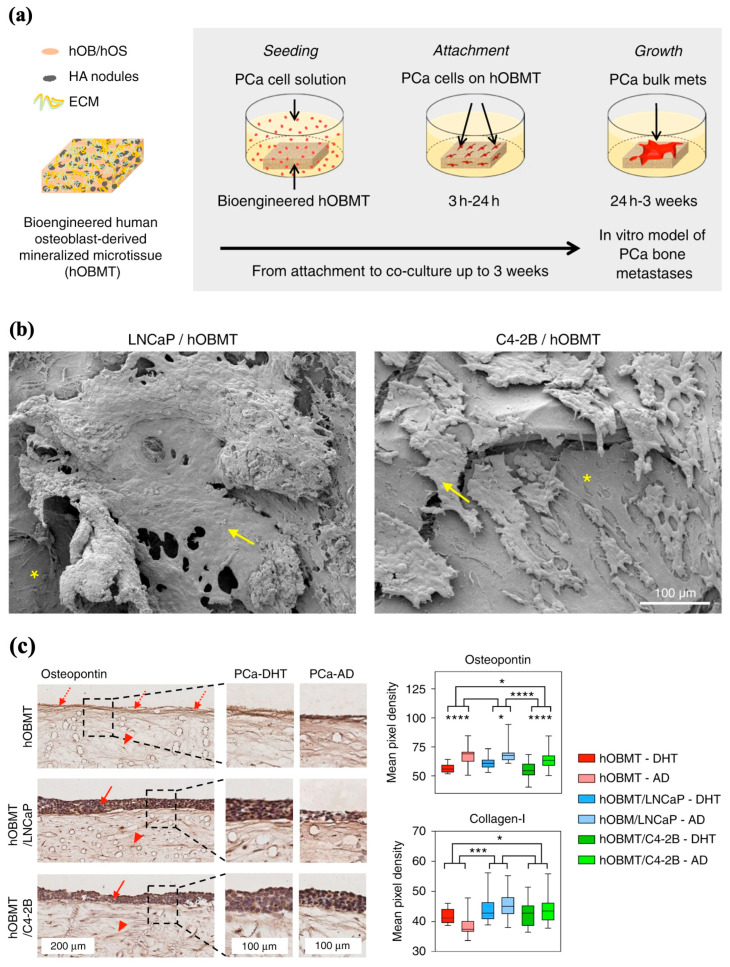
MEW applications in ex vivo disease modeling. (**a**) Diagram representation of human osteoblast-derived mineralized microtissue (hOBMT) cultured with human prostate cell line (PCa), leading to the attachment, proliferation, and metastases of PCa in bone tissue. (**b**) Scanning electron microscopy (SEM) micrographs of attached and micro-metastatic aggregated bone metastatic cell lines (LNCaP and C4-2B) on human osteoblasts. Yellow arrows show cancer cells and asterisks indicate hOBMT. (**c**) Immunohistochemistry staining of osteopontin and collagens-1 of cancer models grown in androgen-present (PCa-DHT) and androgen-deprived conditions (PCa-AD). hOBMT and PCa/hOBMT co-cultures, stained for osteopontin show increased staining towards the surface, for the hOBMT (dashed arrows), and staining in both PCa cell bulk (solid arrows) and the hOBMT (head arrows). (* *p*  <  0.05, *** *p*  <  0.001, **** *p*  <  0.0001). Adapted from Ref. [70].

Bock et al. aimed to develop an in vitro bone metastatic model capable of emulating the cellular and microenvironmental alterations of bone metastases observed in vivo with androgen deprivation [70]. In accordance with a protocol published by the authors, MEW PCL scaffolds were fabricated and subsequently seeded with human-derived preosteoblast cells that underwent osteogenic differentiation to ultimately create a mineralized bone-like microtissue model that imitates healthy bone. This “healthy” model was then co-cultured with prostate cancer cells to produce a final 3D androgen receptor-dependent/independent in vitro representation that exhibited the typical biological features observed of bone metastasis in vivo (Figure 6a,b) [70,71]. In their experiment, when the metastatic models were subjected to androgen deprivation, some cancer cell lines within the metastatic models began to undergo an adaptive regulatory response, synonymous with cancer cell resistance and metastatic progression (Figure 6c). The affirmation of the metastatic model’s responsiveness to androgen interaction, or lack thereof, which is similar to what is seen clinically, indicates the capability of MEW scaffolds to serve as capable substrates to model disease and study treatments [70].

The study of bone pathologies using in vitro 3D models by replicating the bone microenvironment enables investigators to further understand the interactions between blood and immune-related diseases. MEW PCL scaffolds were seeded with human osteoblasts and exposed to osteogenic conditions to develop an in vitro model that mimics the native endosteum [72]. The constructs contained dense ECM with increased expression of both osteogenic markers and endosteal proteins, suggesting the development of an endosteal-like environment. To determine whether the model could replicate endosteum functions, the endosteal 3D scaffold model was co-cultured with hematopoietic stem cells (HSCs) and compared to an HSC-seeded two-dimensional (2D) tissue culture plastic. The 3D endosteal-like construct enhanced HSC expansion, migration, and differentiation, advocating the role of MEW in developing a primitive endosteal model for extension into hematology and immunology applications.

## 6. Cartilage Regeneration

The cartilage is an important structural component of the body. The resilient connective tissue is required to bear loads in joints and intervertebral discs, lubricate joints, and form long bones during development and growth [73]. Cartilage is primarily composed of chondrocytes, specialized cells that produce collagenous fibrils, and extracellular matrix, which are important for maintaining cartilaginous tissues. There are three types of cartilage: hyaline, fibrocartilage, and elastic, all of which contain different proportions of collagen and ECM [74]. Chondrocytes typically function under low oxygen tension because of the limited vascularity of cartilage and rely on anaerobic glycolysis for energy production. Its limited blood supply, along with its limited mobility within the densely packed ECM, poses difficulties for cartilage repair and regeneration. Thus, treatment methods have focused on cartilage tissue engineering to create biologically compatible constructs that can support durable tissue repair systems.

### 6.1. Articular Cartilage

The repair of articular cartilage (AC), vital for bone articulation and vertical load transmission, has been of particular interest, given its limited capacity for healing and repair [75]. The AC is composed of a dense extracellular matrix composed of water, mostly type II collagen, and proteoglycans, with a sparse distribution of chondrocytes. The tissue is composed of three zones defined by the orientation of the collagen fibrils. Zone 1, also known as the thin superficial (tangential) zone, makes up approximately 10 to 20% of tissue thickness and consists of collagen fibers packed parallel to the articular surface. This zone is mainly packed with type II and IX collagen and contains many flattened chondrocytes that help protect and maintain deeper layers. Immediately deep to this layer is the transitional zone, which contains thick collagen fibrils arranged in an oblique fashion. Its composition and organization help provide initial resistance to compressive forces. The deepest zone contains collagen fibers arranged perpendicular to the articular surface and the highest proteoglycan content, which provides the greatest resistance to compressive stress. Homeostasis of the vast ECM metabolism is therefore important for cartilage turnover. However, because the dense ECM is sparely populated with chondrocytes, osteochondral trauma, defects, and disease processes are devastating.

**Figure 7:**
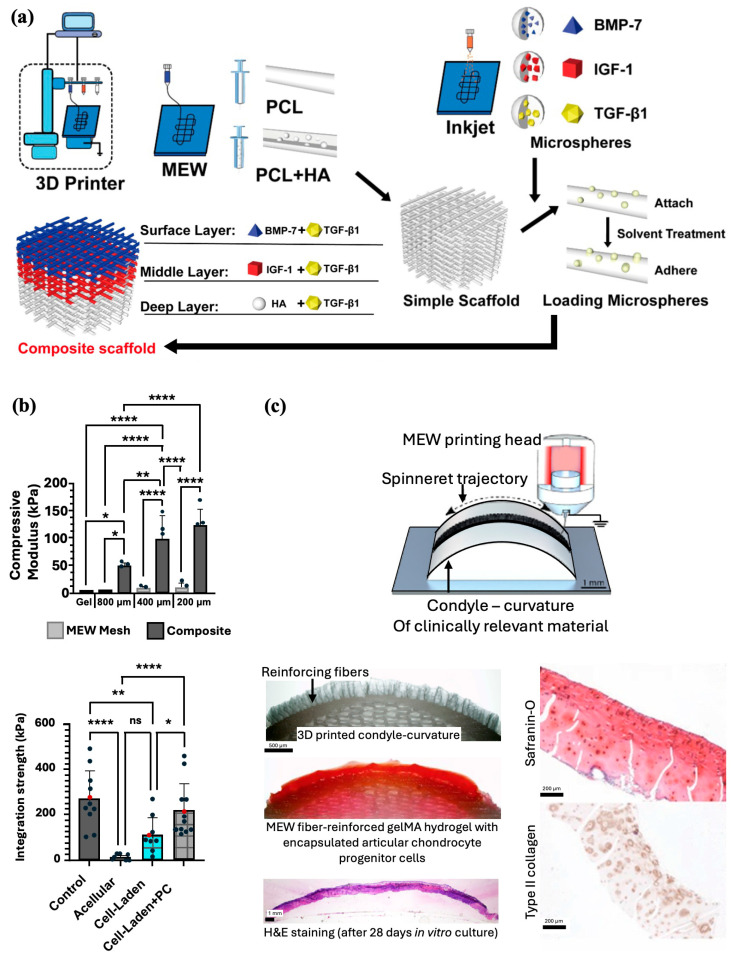
MEW scaffolds fabrication methods for cartilage regeneration. (**a**) Combination of MEW and Inkjet printing to prepare bioactive multilayered composite scaffolds. Adapted from Ref [77]. (**b**) MEW composites made by PCL meshes with varied interfiber spacing and filled with hydrogel formulation resulted in an increased compressive moduli and integration strength (* *p* < 0.05, ** *p* < 0.01, **** *p* < 0.0001). Reproduced with permission from Ref. [79]. Copyright 2021 IOP. (**c**) Fabrication and in vivo implantation of PCL MEW microfiber-reinforced hydrogel that mimics the contour and shape of an articulating joint. Adapted from Ref [84].

With the rise of tissue engineering, there has been increasing interest in the use of scaffolds to create biologically similar 3D constructs capable of providing a mechanically stable environment conducive to chondrocyte regeneration [76]. Given the stratification of cartilage layers, replication of the three zones is imperative to retain natural cartilage function. With MEW’s ability to extrude and stretch thick fibers to reduce their thickness, this technology is valuable for its ability to more precisely print fibers that reflect the complexity of the tissue structures. By combining Inkjet printing, which can be used to integrate bioactive materials into composites with MEW, Han et al. created PCL and hydroxyapatite (PCL/HA) scaffolds that could be implanted into a cartilage injury site in rabbit femoral condyles (Figure 7a). The researchers have developed PCL and PCL/HA scaffolds and attached microspheres containing transforming growth factor beta 1 (TGF-β1), bone morphogenic protein-7 (BMP-7), Insulin-like growth factor 1 (IGF-1), and HA to these scaffolds. Tissue evaluation of the injury site showed that the composite scaffold formed a smooth cartilage on the surface at 12 weeks and integrated well with the surrounding tissue [77]. The group reported similar success in a second study by implanting a gelatin/poly(lactic-co-glycolic acid) composite scaffold integrated with TGF-β1 and HA into a rabbit intercondylar cartilage injury site [78]. Similar to the first study, placement of the composite led to coverage of the injured area by new cartilage and integration with the surrounding tissue. Further histological evaluation confirmed enhanced cartilage repair and normal morphology of cartilage tissue at the injury site 12 and 24 weeks post-surgery.

While initial advances in cartilage engineering have focused on the use of hydrogels, given their cell-friendly environment and lubrification properties, their mechanical strength and integrity limit their use in an otherwise mechanically driven environment [76]. Thus, multi-material architectures have gained interest by simultaneously optimizing cell growth and replicating the structural features of native cartilage. Galarraga et al. found that norbornene-modified hyaluronic hydrogels reinforced with MEW PCL mesh led to a significant increase (50 times) in the compressive modulus of the composite compared to the hydrogel alone (Figure 7b) [79]. Furthermore, composites seeded with mesenchymal stem cells (MSCs) exhibited similarly high cell viability and increased aggrecan and type II collagen expression, suggesting that the PCL meshes did not negatively impact the synthesis and deposition of ECM. When press-fitted into cartilage rings ex vivo, MSC-laden composites pre-cultured in chondrogenic media demonstrated the highest integration strengths, intimate contact with surrounding cartilage, and increased staining of glycosaminoglycans (GAGs) and collagen type II. Overall, combining MEW meshes with hydrogels permits the manipulation of composite mechanical properties to better mimic the natural superficial cartilage environment and enhance cartilage regeneration.

However, within the human body, cartilage is composed of multiple layers with distinct physiological roles. The zonal variation in the cartilage provides an impressive ability to resist compressive forces and minimize friction. Castilho et al. demonstrated that a bi-layered cell-laden hydrogel construct reinforced with PCL microfibers could functionally mimic the superficial tangential, middle, and deep zones found in the native cartilage. In addition, when seeded with chondrocytes derived from healthy equine joints, composite scaffolds demonstrated high cell viability and the ability to form new cartilage under mechanical stimulation without TGF-β1 supplementation [80].

One specific challenge in implementing these scaffolds is the fixation of full-thickness cartilage defects. Previously, four fixation approaches were explored in human cadaveric knees: press-fitting, suturing with an overlaying periosteum, fibrin sealant, and the use of an absorbable mesh anchored with bone [81]. However, given the complex loads experienced within a live model, these approaches often prove difficult to secure within the defect. Galarraga et al. utilized their previously described MEW-NorHA composites to investigate the impact of culturing these implants with adult porcine MSCs and employing different surgical fixation methods on their fixation efficacy in a porcine model of cartilage damage [81]. Two fixation techniques were employed: affixing the composites to the underlying subchondral bone using bioresorbable pins, and fixation using fibrin glue. Arthroscopic observations 12 weeks post-surgery demonstrated that both pinned and glued composites showed heterogeneous defect filling, although the latter facilitated more complete defect filling. Macroscopic evaluation revealed that the pin volume impeded the complete filling of the defects. Mechanical testing of tissues within defects showed that the average compressive modulus of repaired cartilage across all experimental groups was inferior to that of native cartilage. Given these findings, further improvements in the engineered cartilage constructs and fixation methods must be considered.

Therefore, scaffold-free engineering strategies hold promise in cartilage repair. These strategies aim to generate a high-density cell population via self-assembly processes, which can often produce a more hyaline-like matrix than standard implants [82]. Dufour et al. combined MEW and Inkjet bioprinting by ink-jetting MSCs into MEW PCL box-like scaffolds with the aim of forming a network that can guide chondrogenic organization while providing mechanical reinforcement [83]. Once seeded into the MEW microchambers, the cells self-assembled into spheroids and were allowed to grow and fuse with neighboring spheroids. After 21 days in culture, tissue aggregates were found to have grown over the surface and through the microchambers with a strong hyaline-like composition and GAGs content approaching that of the native cartilage. Moreover, the organization of the tissue network closely resembled that of the articular cartilage of porcine synovial joints, conveying a three-layer organization typical of juvenile cartilage. This hybrid approach of bioprinting cells onto MEW scaffolds to produce recapitulated cartilage tissue has the potential to create biomimetic grafts for joint resurfacing.

More on joint resurfacing, Peiffer et al. aimed to improve cartilage repair by translating fabricated structures from flat to more anatomically and biologically relevant surfaces and contours [84]. MEW PCL fibers were printed directly onto fabricated clinically relevant curved surfaces from conductive gelatin methacrylate (gelMA), aluminum, and nonconductive PCL, and magnesium phosphate (MgP) materials (Figure 7c). Accurate deposition onto curved surfaces was found to be successful with low conductive substrates being more conducive for resurfacing anatomically relevant surfaces with MEW fibers. Furthermore, the group confirmed the application by resurfacing a PCL scaffold approximating the native geometry of a human femur with a microfiber-reinforced articular cartilage-resident chondroprogenitor cells (ACPC)-laden gelMA hydrogel. After 28 days in culture, the composite exhibited significantly improved mechanical properties, maintained its shape, and supported the formation of new cartilage.

A separate study evaluating the potential of MEW scaffolds for acetabular labrum restoration reported similar results when determining the effect of decreased fiber spacing on the mechanical properties of PCL scaffolds. However, interestingly, these scaffolds were unable to replicate the mechanical properties of native acetabular labrum tissue despite exhibiting promising biocompatibility with primary labrum cells. Furthermore, scaffolds with a waved architecture were fabricated to match the hyperelastic behavior of labrum tissue and even withstand physiological strain under mechanical testing. Unfortunately, these scaffolds could not promote a labrum cell response under dynamic loading [85]. Overall, using MEW to construct scaffolds of varying architectures recapitulates the mechanical properties of the labrum and allows the migration of cells to grow along the scaffold, highlighting its potential as a graft candidate.

### 6.2. MEW Potential in Regenerating the Osteochondral Interface

Apart from singular bone defects, studying the regenerative potential of lesions involving both articular cartilage and subchondral bone is also of notable importance, considering the anisotropic nature of osteochondral tissue. Given the structural complexity of the osteochondral junction, Diloksumpan et al. employed MEW to print a 3D construct that served as a secure interface between two mechanically distinct materials. Once PCL meshes were fabricated using MEW, the authors utilized pneumatic extrusion-based 3D printing to anchor a calcium phosphate-based bioceramic “bone” ink onto one side of the mesh and embedded an articular cartilage chondroprogenitor cell-laden gelatin hydrogel onto the opposite side. Mechanical analysis of the MEW microfiber-reinforced composites revealed a significant increase in the interfacial strength by over sixfold compared to composites that were created with the bioceramic ink and hydrogel alone. Moreover, reinforcement of the chondral hydrogel with MEW meshes bolstered the compressive strength of the compartment to that of the native cartilage. Most importantly, these multiscale composites were conducive to cartilage-like matrix deposition and osteogenesis within the osteal and chondral compartments, which further enhanced the strength at the osteochondral interface [86].

**Figure 8:**
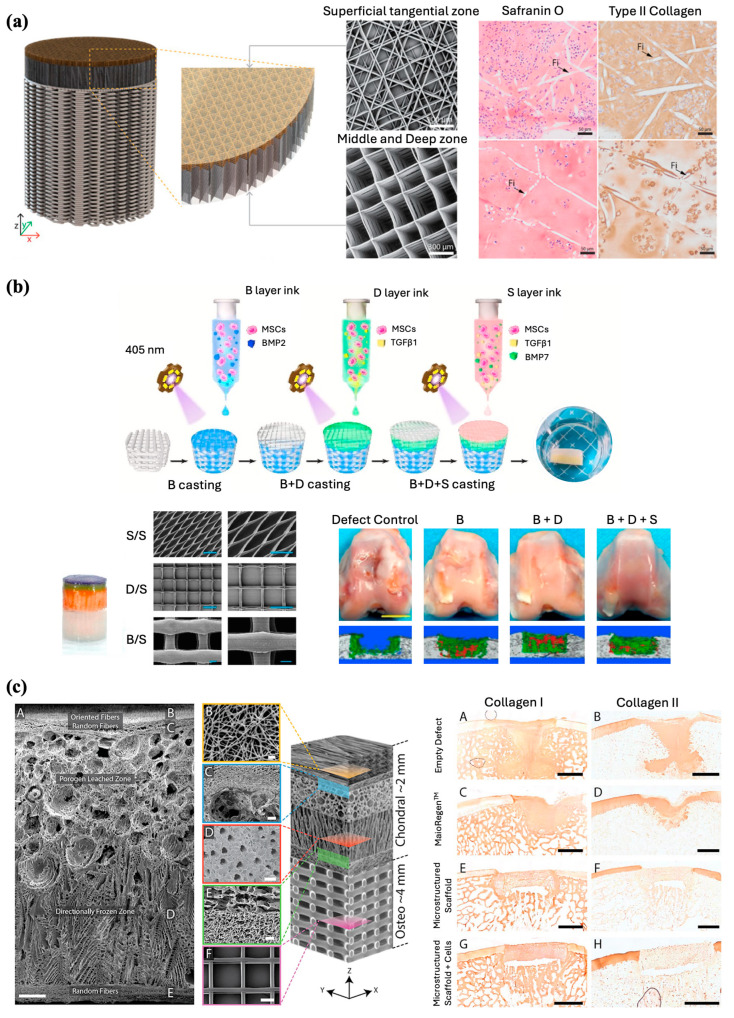
Fabrication of multilayered scaffolds for osteochondral interface regeneration. (**a**) Illustration and hematoxylin and eosin (H&E) staining of bi-layered bone/cartilage scaffold containing horizontal and vertical reinforcing fibers through the cartilage phase. Adapted from Ref. [87]. (**b**) Schematic diagram demonstrating MEW fabrication process of a bioactive tri-layered scaffold and subsequent osteochondral defect regeneration in vivo (B = subchondral bone layer, D = deep cartilage layer, S = superficial cartilage layer). SEM images of the fibrous networks (scale bar 200 μm). Gross observations and micro-CT images of defect repair at 24 weeks post-surgery for the composite scaffolds (Scale bar 5 mm). Reproduced with permission from Ref. [89]. Copyright 2021 Elsevier. (**c**) Scanning electron micrographs of a zonal microstructured scaffold (Scale bars for images A, B, C, D, E, and F are 250, 10, 50, 100, 25, and 100 μm, respectively), and immunohistochemistry staining for collagen type 1 and type II in the explants (Scale bars 2 mm). Adapted from Ref. [88].

Ruitjer et al. constructed a multilayered cartilage implant of hydrogels reinforced with embedded PCL fibers using MEW with extrusion-based bioprinting of ceramic and hydrogel. This fabrication process resulted in enhanced compressive and shear properties approaching those of the native cartilage tissue. An osteochondral implant consisting of three different layers was fabricated with 50 layers of PCL MEW fibers infused with 8% gelMA. The middle and deep zones consisted of box-like MEW PCL fibers with an inter-fiber distance of 300 mm, whereas the superficial tangential zone consisted of fibers deposited in a laydown pattern 0°-45°-90°-135°. The complex shear and compressive moduli of the bi-layered reinforced structures showed a notable increase compared to the boxed-reinforced and non-reinforced cell-laden hydrogels (Figure 8a). A high level of complexity and biomimicry of the construct was achieved with high resolution, high reproducibility, and small diameters of the MEW fibers [87].

Similarly, Steele et al. engineered a porous microstructured PCL scaffold using a combination of MEW, electrospinning, porogen leaching, and directional freezing techniques (Figure 8c). The resulting constructs closely imitated the multizonal structure and mechanical properties of articular cartilage [88]. The three zones consisted of a superficial zone with aligned electrospun fibers, a porogen-leached intermediate zone, and a directionally frozen deep zone, which would further mimic the mechanical gradients across the articular cartilage. In vitro investigations demonstrated that the scaffold effectively supported chondrocytes and facilitated matrix deposition. Subsequently, the viability of the scaffolds was assessed using a porcine osteochondral defect model. Macroscopic, histological, and micro-CT analyses indicated that the microstructured PCL scaffold supported matrix deposition and osteointegration, while maintaining its integrity within the defect after 6 months.

With the aim of osteochondral tissue regeneration, Qiao et al. used a similar principle and developed a tri-layered scaffold inspired by the superficial cartilage, deep cartilage, and subchondral bone layers of the native osteochondral tissue. A combined PCL-poly (ethylene glycol) (PCEC) polymer was used to fabricate the scaffold via MEW, with certain parameters modulated for each layer to best mimic the microarchitecture of each natural osteochondral tissue compartment. All three compartments were subsequently cast with MSC-laden hydrogels containing zone-specific growth factors to aid in cell differentiation (Figure 8b). In vitro assessment of the scaffold showed cartilage zone-specific markers expression and collagen II deposition within the superficial and deep layers and osteogenesis-related collagen I deposition within the bone layer near the osteochondral interface of the scaffold. Analysis of the mechanical properties to evaluate the load-bearing capacity revealed that the MEW PCEC-reinforced hydrogel composites outperformed both pure fiber scaffolds and hydrogels alone. Macroscopic observations of explants harvested from rabbit osteochondral defects demonstrated complete filling and excellent integration of defects. Histological staining and micro-CT analysis further elucidated simultaneous regeneration of neo-cartilage and subchondral bone with a continuous transition noted to be formed between the two. Researchers also noted the regeneration of a more lubricated and wear-resistant cartilage surface within the in vivo model, credited to the inclusion of a superficial cartilage-inspired layer in their construct [89]. These studies further highlight the importance of MEW in osteoregenerative applications, as MEW scaffolds promote mechanical stabilization for improved surgical handling and implantation while also facilitating the development of a spatially customizable construct that can integrate multiple tissues [86,89].

Table 3 highlights key studies leveraging MEW scaffolds for bone and cartilage regeneration. It outlines how architectural design parameters, integration with complementary 3D printing techniques, and incorporation of biomimetic materials influence scaffold performance. The table provides insight into scaffold composition, fabrication strategies, and observed outcomes, offering a comparative overview of advancements in MEW-based tissue engineering for both bone and cartilage applications.

### 6.3. Meniscus

The meniscus is a fibrocartilaginous tissue that plays an important role in providing support to the surrounding articular cartilage within joint cavities. In the human body, menisci can be found in the knee, wrist, acromioclavicular, sternoclavicular, and temporomandibular joints. Generally, the term “meniscus” is colloquially used to refer to tissue found in the knee joint. The tissue comprises of two components found between the femoral condyle and tibial plateau, which disperse friction and facilitate weight distribution within the joint. Tissue vascularization gradually decreases throughout childhood and generally becomes avascularized in adulthood, compromising the healing capacity of each area [91]. Destruction of this tissue can result in gradual deterioration of the surrounding articular cartilage, leading to the development of osteoarthritis [92]. Given the significant challenge of healing defects, current treatment methods have only been effective in treating lesions located within regions of minimal vascularity or partial meniscectomy, which may lead to long-term osteoarthritic changes [93].

**Figure 9:**
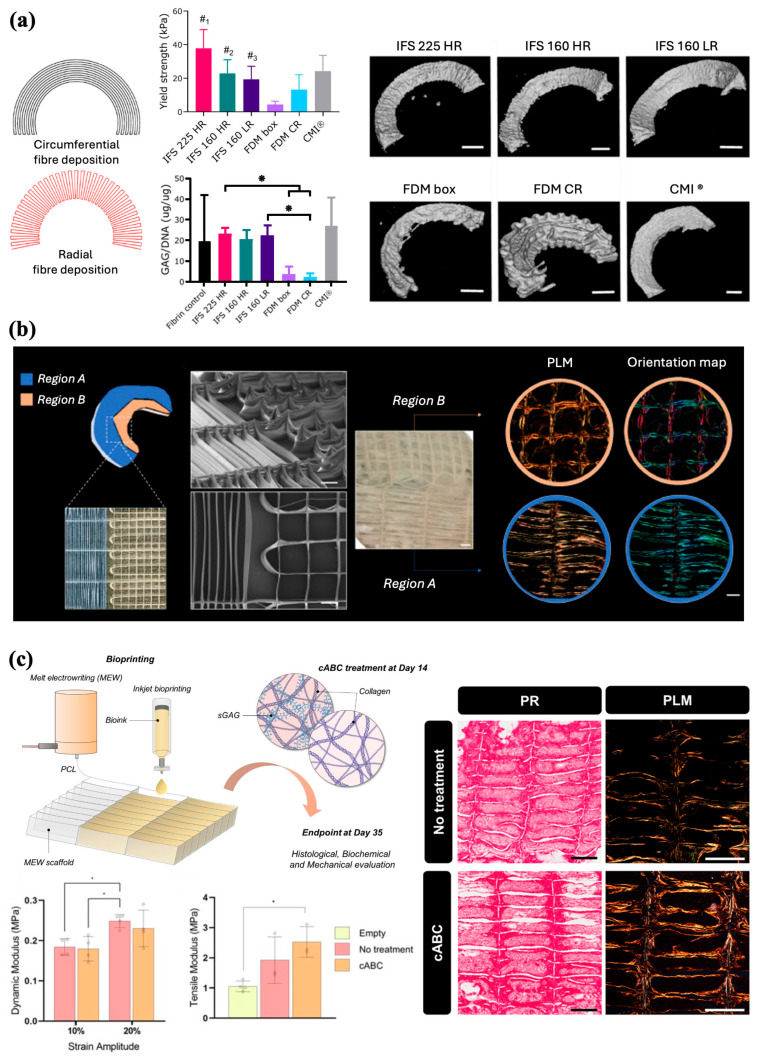
MEW scaffolds architecture for tissue organization and meniscus repair. (**a**) Scaffold design inspired by native fiber architecture with circumferential and radial fibers. Yield strength, proteoglycans production, and micro-CT images after 28 days of co-culture (*p < 0.05, #1, compared to all groups; #2, compared to all groups except CMI^®^ and FDM CR; #3, compared to FDM box and IFS 225 HR) (Scale bar 2 mm). Adapted from Ref. [95]. (**b**) Spatially defined architecture organization and SEM images of the meniscus-inspired scaffolds (Scale bar 500 µm). Bright field image (Scale bar 800 µm) of the scaffold after 4 weeks of in vitro culture, and polarized light and color map imaging of the collagen fibre distributions (Scale bar 400 µm). Reproduced with permission from Ref. [96]. Copyright 2023 Elsevier. (**c**) Combination of MEW and inkjet bioprinting for the fabrication of scaffolds with defined pore architecture and collagen fibers parallel to the long axis of the scaffold pores. Biomechanical properties and collagen organization of the engineered tissues following 5 weeks of in vitro culture (* *p* < 0.05). Adapted from Ref. [98].

Current approaches to meniscus repair include replacement with a collagen meniscus implant (CMI) or total allograft transplantation [94]. Limitations of either option, however, include the necessity to use a robust scaffold capable of providing weight-bearing support and the costly handling of allografts. Therefore, tissue engineered biocomposite implants provide an alternative solution by expanding the limited mechanical properties of current implants. Although, FDM has been previously used to fabricate meniscus scaffolds capable of being seeded with mesenchymal progenitor cells, in vivo implantation proves difficult given the stiffness of the implant. MEW has previously been shown in articular cartilage to have high reproducibility with less stiffness to reduce damage to the surrounding tissues. Korpershoek et al. compared the feasibility of a wedge-shaped meniscus scaffold printed with MEW and its compressive properties with current CMI and FDM scaffolds (Figure 9a). In terms of mechanical properties, MEW scaffolds demonstrated greater stiffness than CMI and a higher yield point than FDM scaffolds. While retaining its shape, the MEW scaffold also maintained cell viability after 28 days in culture and produced a basal level of GAGs comparable to that of the controls. Moreover, the high precision and tailorability of MEW technology enable the production of scaffolds that closely mimic the circumferential and radial fiber organization seen in the native meniscus. Considering the structural and functional capabilities of MEW scaffolds observed in this study, the potential for translation as a meniscus replacement treatment option remains entirely feasible [95].

Barcelo et al. employed the tailorability of MEW to replicate the anisotropic nature of the collagen fiber network within meniscal tissue [96]. PCL scaffolds with varying internal architectures and fiber diameters were fabricated via MEW by modifying the aspect ratio of the scaffold pores (Figure 9b). A cell-laden bioink was subsequently deposited into the scaffolds using inkjet bioprinting to evaluate the spatial organization of the collagen network produced by the cells within the scaffolds. For both FDM- and MEW-printed scaffolds, modifying the structural organization of the pores in which cells were physically confined allowed for control of the direction in which the collagen network was laid down by cells. Compared with FDM, MEW scaffolds had compressive mechanical properties comparable to those of native tissue and were able to support the development of fibrocartilaginous tissue. However, when assessing tensile properties, MEW scaffolds have noticeably less strength than human meniscus, which often displays high tensile stiffness, given the circumferential organization of collagen fibers [97]. To improve the mechanical properties of these MEW-bioprinted meniscal tissues, Barcelo et al. treated scaffolds with chondroitinase ABC (cABC), an enzymatic treatment previously shown to enhance collagen fiber maturation [98]. Polarized light microscopy showed that enzymatic treatment supported a more collagen-rich matrix, although it did not influence collagen network alignment. cABC treatment also increased the tensile modulus compared to that of the empty MEW scaffold. Therefore, the inclusion of enzymatic treatments provides a biofabrication strategy to construct a more mechanically functional scaffold.

The customizability afforded by MEW further enabled Barcelo et al. to scale up the biofabrication of scaffolds to engineer large wedge-shaped meniscus-like tissues cultured with zone-specific meniscus progenitor cells (MPCs) to engineer constructs that could further mimic the complex architecture of native tissues (Figure 9c) [99]. Spatially depositing MPCs obtained from either the outer (oMPCs) and inner (iMPCs) zones of native meniscal tissues through anisotropic pore networks demonstrated the capacity of cells to generate fibrocartilage-like tissue organized within zones. Culturing previously developed MEW PCL scaffolds with MPCs along with chondrogenic culture [96] exhibited robust chondrogenesis with preferential alignment of collagen fibers, following the physical boundaries imposed by the scaffold. Light staining confirmed that oMPCs expressed the lowest level of type II collagen representative of the outer region of the meniscus; conversely, iMPCs expressed the lowest level of type I collagen, reflecting the inner zonal phenotype of the meniscus. The MEW scaffolds were then scaled up iteratively to mimic the wedge-shaped tissue structure. Culturing the scaffold with iMPCs and oMPCs into distinct regions within the scaffold generated a type I collagen right fibrocartilage-like matrix, similar to the native meniscal composition. Overall, the capability of using a novel biofabrication framework to engineer structurally organized meniscal grafts underscores the possibility for regenerative medicine applications and potential treatments for meniscal repair.

## 7. Ligament and Tendon Regeneration

Tendons are composed of connective tissue that acts as a mechanical bridge transmitting muscle strength to the bones and joints. They play an important mechanical role in the movement and maintenance of body posture. This is made possible by their high tensile strength, rigidity, and stiffness. Tendons consist of tenoblasts and tenocytes, which are specialized fibroblasts that produce ECM-containing collagen (types I, II, and III), elastin, proteoglycans, and glycoproteins among other proteins. The organization of collagen fibers parallel to each other and the tendon axis is crucial to their function, with the bundles exhibiting a wavy pattern and periodic changes of direction known as crimps. Understanding this important quality is crucial for developing scaffolds and engineering tissues that closely mimic native tendons [100]. Similarly, ligaments consist of closely packed, parallel collagen fibers that have different degrees of undulation along the axis of each fiber at a resting length, and they also exhibit crimps [101]. Furthermore, they share the same cellular composition of fibroblasts and protein-rich ECM, which allows them to swell with water, which acts as a lubricant. The different mechanical forces acting on each specific tendon or ligament have a dynamic effect on establishing its specific protein composition. However, tendons are unique in that bundles of fibers are crosslinked to each other by short fibrils, which bolster their tensile strength.

Another important feature of both tendons and ligaments is the limited blood supply passing through the tissue; instead, they rely on diffusion across cells from blood vessels arising from the tendon sheath and the periosteum, respectively [102]. Although this improves their structural integrity, it hinders their regenerative ability when injured or damaged. Moreover, ligament and tendon injuries account for 50% of musculoskeletal injuries, with an estimated annual cost of over $40 billion, notwithstanding the impact on patients’ quality of life. Injuries that result in complete tear of tendons or ligaments often require surgical treatment, which while successfully treating the patient, has been associated with donor-site morbidity, especially in cases of large tears that lead to inferior scar tissue formation and are more prone to re-rupture. In addition to surgery, current treatment options focus on biophysical stimulation (physical therapy, cryotherapy, magnetic fields, and ultrasound), the injection of growth factors into the tissue (most commonly platelet-rich plasma), and tissue engineering using stem cells and scaffolds [103]. MEW has the potential to further advance this area of intervention, especially in difficult cases with poor healing and high risk of re-rupture.

### 7.1. Effect of Pore Architecture on Cell Behavior

Understanding that scaffolds used in ligament and tendon regeneration must be able to support cell migration and the colonization from surrounding tissues for remodeling of the damaged ligament or tendon, it is imperative to evaluate the influence of scaffold pore parameters on cells [104]. In addition to assessing BMSC behavior as described earlier, Han et al. also explored the impact of 50, 100, 200, 300, and 400 µm pore-sized scaffold groups on the adhesion, proliferation, and differentiation of rabbit tendon stem cells (TCs). After 14 days of culture, fluorescence imaging of the TC-seeded scaffolds revealed low adhesion and proliferation in larger pores, indicating incomplete filling. In addition, TCs located within the center of the pores were thinner than the cells directly attached to the fibers. Further analysis of cell proliferation via cell counting kit-8 (CCK-8) testing after 21 days of culture revealed that the 200 and 300 µm scaffold groups provided the best favorable environment for cell proliferation, with a slight edge to the 300 µm scaffolds. All scaffold groups exhibited good tendon stem cell viability [44].

Von Witzleben took further advantage of the tailorability of MEW through a more complex alteration of scaffold fiber and pore designs with the aim of assessing cell alignment and migration for ligament or tendon reconstruction. Five different PCL scaffold groups were fabricated with various fiber diameters and pore structures. Three scaffolds were produced with the following fiber diameters: thin fibers (5 µm), thick fibers (15 µm), and mixed thin and thick fibers (both 5 and 15 µm). The last two scaffolds were designed to have a rhombohedral pore geometry, with the smallest angle being either 30° or 70°. All five scaffolds were then cultured with primary neonatal human dermal fibroblasts to evaluate their coverage and alignment. After 14 days in culture, all scaffold groups were found to have good cell coverage, with a partially dense layer of cells spanning the pores. In terms of cell alignment, scaffolds with mixed and 30° fiber patterns demonstrated a visually distinct vertical orientation of cells, with the 30° fiber pattern exhibiting the greatest amplitude in oriented cell count. To further mimic the biological and biomechanical characteristics of the native ligament/tendon tissue, these two superior fiber architectures were subsequently combined with a mechanically advantageous embroidered structure. The mixed and 30° fiber patterns were individually printed on top of separate embroidered structures, and the composites were then coated with collagen to enhance biocompatibility. Mechanical analysis demonstrated that the fortification of the MEW scaffolds with embroidered structures led to an improvement in mechanical properties approaching that of native anterior cruciate ligament (ACL) tissue. More specifically, the Young’s modulus of the composites increased compared to that of pure MEW scaffolds yet was still found to be approximately half that of ACL tissue. Moreover, the addition of the embroidered structure improved the stiffness of the composites as well, which was not significantly different from that of the ACL. Finally, cell alignment was re-evaluated for these new scaffold groups; however, the authors elected to seed scaffolds with adipose tissue-derived mesenchymal stem cells (AT-MSC), which are a more representative cell type for ligament and tendon tissue. Interestingly, the addition of MEW fibers to the embroidered structures promoted better cell migration and distribution throughout the surface of the construct. Additionally, while all scaffolds showed a preferred cell orientation angle of 90°, for those involving embroidered structures, the inclusion of the 30° fiber pattern resulted in the highest visible cell alignment. Collectively, although the results illustrated above are somewhat far from reproducing the properties of native ligament/tendon tissue, the techniques and outcomes of this investigation offer an intriguing foundation for the development of a potential synthetic substitute for ligament and tendon reconstruction [104].

### 7.2. Architectural Design: Crimped, Aligned or Sinusoidal

**Figure 10:**
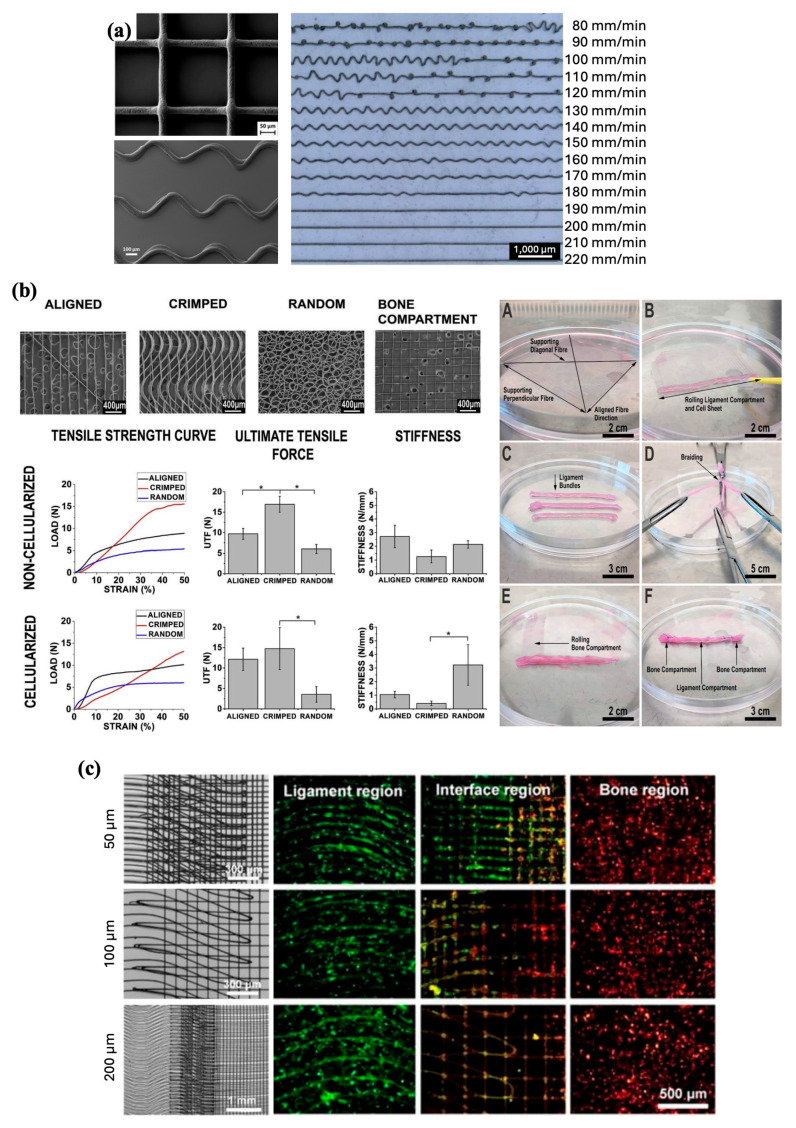
Modulation of fiber morphology for optimized mechanical properties and ligament regeneration. (**a**) Effect of the collector’s translation speed (TS) on the printed fiber morphology of PCLAC. Reproduced with permission from Ref. [106]. Copyright 2018 Elsevier. (**b**) MEW printing of aligned, crimped, and random fibers, and their assembly into a bone-ligament-bone construct, with higher ultimate tensile force in the crimped constructs (* *p* < 0.05). Reproduced with permission from Ref. [107]. Copyright 2020 Elsevier. (**c**) Images of the MEW grid-crimp micropatterns with different preset fiber spacings along with staining of NIH/3T3 cells and Saos-2 cells on the crimp micropattern (ligament), grid micropattern (bone), and the in-between interface region. Reproduced with permission from Ref. [108]. Copyright 2022 IOP.

The “crimped” collagen fibers that comprise tendons play an important role in the biomechanical nature of these tissues. When relaxed, these wavy fibers buckle together; however, during motion, they undergo unbuckling to absorb shock and provide elastic recoil [105]. This behavior explains the “toe region” of the tendon stress-strain curve, where these fibers stretch at low strain. To approximate this mechanical behavior, Hochleitner et al. used melt electrowriting below the critical translation speed to generate scaffolds with sinusoidal fiber patterns using a suitable ultraviolet (UV) cross-linkable polymer, poly(ε-caprolactone-co-acryloyl carbonate) (PCLAC) [106]. In terms of fabrication, a translation speed at approximately 68% CTS allowed printing of sinusoidal fiber patterns with maximum peak-to-peak and minimum-wavelength values observed (Figure 10a). While these scaffolds had wavelengths and peak-to-peak values in ranges not in accordance with native tendons and ligament tissue, tensile load testing of scaffolds revealed that distinct sinusoidal fiber patterns produced mechanical responses corresponding to the ranges studied in ligament and tendon tissue. The significant increase in tensile strength and elasticity, along with creep elimination, is partly attributed to the presence of cross-linking sites within sinusoidal scaffolds formed by UV curing. Collectively, approaches to customizing fiber morphology and printing strategies have qualified MEW scaffolds as promising devices to elicit ligament and tendon regeneration [106].

Given that regulating conductor speed did not adequately control the alignment of the crests and troughs of sinusoidal fibers, Gwiazda et al. studied the effects of topographical guidance on fiber organization and its effects on the alignment behavior of hMSCs. In the context of application to the ACL, three configurations were used: aligned, crimped resembling the ACL organization, and randomly deposited fibers (Figure 10b). On a micro level, they produced optimally aligned fibers with better control over the angle and period of crimping than other methods, allowing for more consistently reproducible results. They also reinforced the idea that stacking or superimposing fibers could overcome the limitation of producing fibers with a spacing or pore diameter of 200 μm, which is related to cell alignment. Although a crimped configuration is naturally seen in ligaments like the ACL, only the aligned scaffolds achieved efficient cellular alignment towards the direction of fibers throughout the entire experiment. On a macro level, they assembled acellularized and cellularized scaffolds into ligament-like braids, and a mineralized bone compartment scaffold was added to each end to create a bone-ligament-bone construct. Testing revealed that cellularization of the scaffolds increased the resilience and elasticity of the construct, but the mechanical properties of the resulting constructs were lower than those of the ligament tissue [107]. However, the mechanical properties of these constructs can be significantly improved by in vivo maturation.

Building upon these micropatterned scaffolds, Xiong et al. developed a MEW-based grid-crimped fiber writing method to mimic the bone-ligament interface to study the effects of fiber patterns on cell alignment and osteogenic activity of osteoblastic/fibroblast co-cultures (Figure 10c). To fabricate the micropatterns, three-stacked layers of PCL crimped fibers were used for the crimped part of the pattern and three stacked layers of PCL straight fibers were used for the grid part. The ligament region was recreated using various crimp angles and fiber spacing within the crimped pattern. Xiong et al. showed that scaffolds with the smallest fiber spacing showed the most significant fibroblast alignment, enabling the fastest migration of both fibroblasts and osteoblast-like cells. A smaller fiber spacing within the triphasic scaffold also favored the interaction of both cell types within the interface region, whereas few cells were observed in the region with greater fiber spacing. Specifically, the scaffold with 50 µm fiber spacing was the most effective in guiding fibroblast orientation, with the cell orientation angle closely matching that of their respective crimp angle. To examine their potential as bone-ligament interface grafts, these triphasic scaffolds containing different fiber spacings were rolled. All scaffold variants showed mechanical qualities like natural bone-ligament articulation, suggesting that tubular scaffold may serve as an appropriate conduit for bone-ligament tissue grafting or engineering [108].

## 8. Skeletal Muscle Regeneration

The body contains over 600 individual skeletal muscles, which make up about 40 percent of total body mass, making them the largest tissue by mass [109]. Skeletal muscles are not only essential for the movement and support of the body but also for regulating body temperature and storage of nutrients. Skeletal muscle is hierarchically organized as bundles of parallel aligned myofibers that differentiate through multinucleated myotubes with diameters in the range of 20–100 μm. Myotubes further differentiate into myofibers, which are covered by a thin layer of connective tissue (endomysium) mostly comprised of laminin and type IV collagen [110]. A key feature of skeletal muscle is the specialized ECM that surrounds it, containing essential proteins, such as collagen, fibrin, and integrins, which are vital for skeletal myogenesis and normal physiological function [111].

Skeletal muscle is well-known for its ability to regenerate new fibers after injury by trauma or as a consequence of disease [112]. However, there is a limiting regenerative threshold of approximately 20% muscle loss since large volumes of muscle loss are not capable of repair without interventional support. Major loss of skeletal muscle with lasting impairments significantly impacts the functionality of the skeletal system and quality of life of patients, and is termed “volumetric muscle loss” [113]. With limited success in the use of autologous muscle transfer due to the increased risk of donor site morbidity and inadequate innervation, along with up to a 10% risk of complications, including infection and necrosis, therapies involving tissue engineering are becoming increasingly important. Biomaterials constructed in a 3D fashion can be used to mimic the properties of the ECM, providing chemical and physical cues to transplanted or host muscle cells to enhance their survival and promote their proliferation and differentiation into mature muscle fibers [113]. MEW offers a potential breakthrough in generating optimal 3D scaffolds to achieve these goals.

### 8.1. Use of MEW in Fabricating Anisotropic Scaffolds to Influence Muscle Cell Behavior

**Figure 11:**
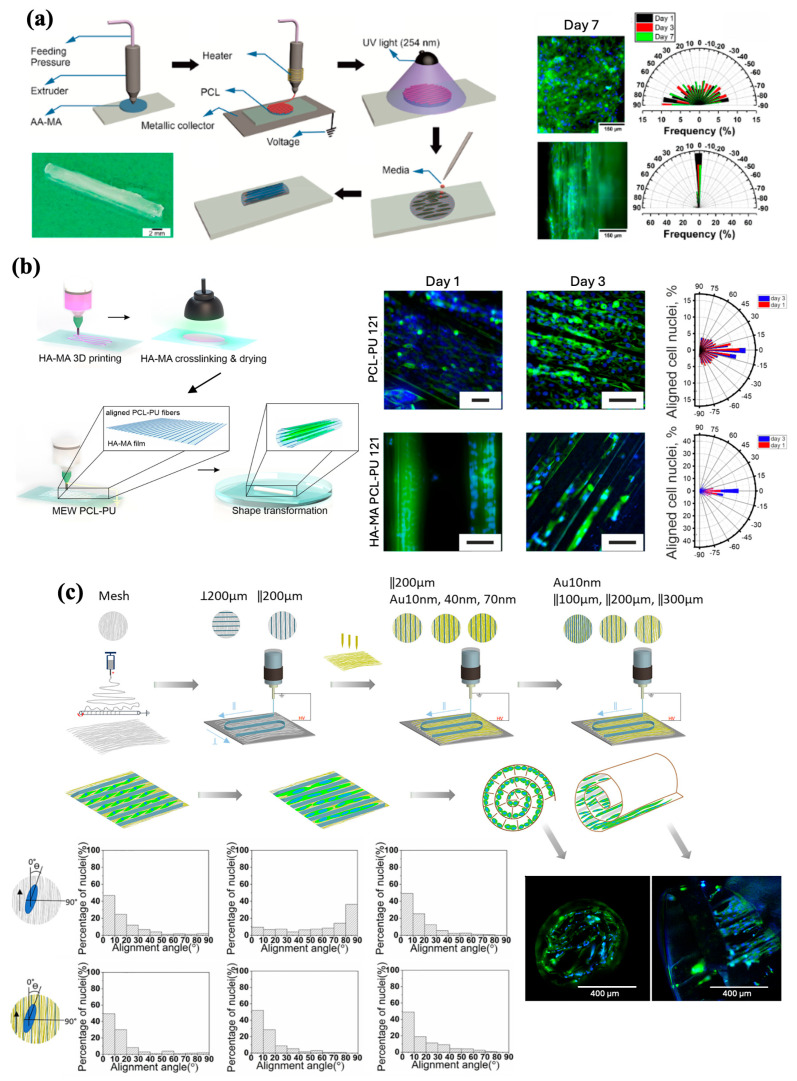
Cellular orientation and encapsulation for muscle tissue engineering. (**a**) Fabrication process of PCL and methacrylated alginate bilayer self-folding tube via MEW and 3D extrusion printing. Myoblasts cultured on the scaffolds showed increased alignment in the direction of the fibers. Reproduced with permission from Ref. [114]. Copyright 2021 American Chemical Society. (**b**) Bi-layered scaffolds with shape transformation capabilities were fabricated using extrusion of methacrylate hyaluronic acid (HA-MA) and MEW deposition of PCL-polyurethane (PCL-PU). Myoblasts encapsulation and nuclei alignment imaging and quantification on scaffolds (Scale bar 100 µm). Reproduced with permission from Ref. [115]. Copyright 2021 American Chemical Society. (**c**) Fabrication of 3D patterned fibrous scaffolds with varied microgrooves spacing. Distribution of nuclei alignment angle after 7 days on different scaffolds with microgroove spacing of 200 μm, including those with Au coating. Reproduced with permission from Ref. [116]. Copyright 2020 Elsevier.

Given that skeletal muscle is composed of aligned fiber bundles arising from the differentiation of myoblasts into long myotubes, recent studies have aimed to target myoblast alignment for anisotropic skeletal muscle myogenesis. Constante et al. developed a self-folding, bilayer scaffold composed of PCL and methacrylated alginate (AA-MA) fabricated via MEW and 3D extrusion printing, respectively (Figure 11a) [114]. Scaffolds were then cultured with a mouse myoblast cell line and exposed to an aqueous solution, which promoted the swelling and self-folding of the scaffolds into tube-like structures. In assessing myoblast biocompatibility, both scaffold groups demonstrated high viability and increased cell proliferation over time; however, the rate of cell proliferation was found to be greater on bilayer scaffolds than on AA-MA controls. More notably, the addition of MEW PCL fibers to the bilayer scaffolds led to a significant increase in the proportion of highly aligned myoblasts relative to the absence of alignment observed in the control scaffolds. Although cell alignment on bilayer scaffolds decreased as the culture progressed, this was likely attributed to a reduction in the adhesion of myoblast membrane proteins to alginate due to its high hydrophilicity [114].

To overcome this drawback, the group sought to improve the shape-morphing bilayer scaffold to further enhance its potential as a substrate for myogenesis [115]. Extrusion 3D printing of methacrylate hyaluronic acid (HA-MA) and uniaxial MEW deposition of PCL-polyurethane (PCL-PU) were used to print the two layers of the modified scaffold (Figure 11b). Intriguingly, while myoblast culture of HA-MA scaffolds resulted in no alignment and seeding of PCL-PU scaffolds only showed growth within pores, myoblast-laden HA-MA—PCL-PU bilayers exhibited adherence of cells to the MEW fibers, as well as formation of cell bridges within the pores. Regardless of the inclusion of a HA-MA layer, myoblast interaction with MEW fibers facilitated their alignment in the direction of the fibers, even of cells that grew within the pores. However, the presence of HA-MA within the bilayer supported greater cell adhesion and attachment to the MEW layer, leading to a higher degree of cell alignment [115].

Zhang and colleagues also looked to utilize the micro-architectural advantages MEW fabrication offers by developing hierarchically organized scaffolds with anisotropic topography conducive for myogenesis (Figure 11c) [116]. Using aligned PCL nanofiber meshes fabricated via solution electrospinning as a base, microgroove fibers were printed by MEW in directions perpendicular or parallel to the aligned nanofibers, and the scaffolds were seeded with a myoblast cell line. While the nanofibers were critical in achieving myoblast cell alignment, the parallel orientation of the MEW PCL microgrooves significantly increased myotube length and enhanced maturation compared to scaffolds with perpendicularly oriented fibers. Subsequently, MEW microgrooves were printed in parallel on gold-coated nanofiber meshes with various spacings between microgrooves (100, 200, or 300 µm) to determine their effect on myotube formation. Analysis of cell morphologies in these experimental groups revealed that 200 µm spacing resulted in the best combination of orientation and size compared to smaller and larger spacing patterns, with myotubes on 200 µm spaced scaffolds demonstrating significantly higher average length and closer orientation to the axial direction of the nanofibers. These results indicate for a primary role in myoblast cell alignment, which is a necessary checkpoint in the formation of myotubes and muscle fiber bundles. In turn, with the assistance of MEW, the possibility of fabricating anisotropic, topographically appropriate scaffolds holds potential to enable the growth of oriented muscle microtissue, an early step towards the regeneration of functional skeletal muscle [114,115,116].

### 8.2. Stimulation of Enhanced MEW-Based Scaffolds for Skeletal Muscle Regeneration

In addition to attempting to replicate the anisotropic architecture of native skeletal muscles, researchers have also considered targeting the electrical and mechanical responsiveness of muscle tissues [117,118]. Zhang et al. successfully fabricated anisotropic, gold-coated conductive scaffolds composed of aligned nanofibers and MEW-printed microgrooves and found a positive relationship between gold coating thickness and both myoblast alignment and myosin heavy chain expression [116]. In a subsequent study, the group proposed applying electrical stimulation to the conductive scaffolds as a method of further promoting myoblast alignment and maturation [117]. PCL microgrooves with 200 µm spacing were MEW-printed on top of PCL nanofiber meshes coated with gold nanoparticles, and the conductive construct was seeded with myoblasts and subjected to electrical stimulation at 1, 2, and 3 volts (V). Cell morphology and differentiation analysis revealed a profound effect of electrical stimulation on myotube development. Compared to non-stimulated scaffolds, significant elongation of myotubes was observed with 1, 2, and 3 V stimulation, and a significant increase in myotube average width was observed with 2 and 3 V. Moreover, a stimulation-dependent trend was observed, with increasing amounts of electrical stimulation leading to significant increases in the ratio of the total myotube area to the total image area under microscopic fluorescence imaging analysis. Altogether, electrical stimulation of these anisotropic electroactive scaffolds showed reasonable efficacy in promoting myotube formation and maturation, possibly restoring damaged muscle tissue [117]. Given the recent increase in electroactive polymer applications and in an effort to further realize the potential of MEW capabilities, future studies may examine the possibility of embedding gold nanoparticles with PCL polymer to form a conductive polymer blend and evaluate its impact on facilitating myogenesis via electrical stimulation [117,119].

On a separate note, magnetic stimulation of magnetized MEW scaffolds has been studied as a method of influencing the mechanical responsiveness of muscle cells. In their approach, Cedillo-Servin et al. created a novel melt-blended polymer of PCL and magnetized graphene nanoplatelets (PCL/rGNP@), the latter being synthesized from the deposition of iron oxide nanoparticles on oxidized graphene nanoplatelets [118]. Composite scaffolds with controlled hexagonal microarchitectures were then fabricated using MEW with increasing magnetic profiles based on the weight percentage of rGNP@ particles added (2, 10, and 20%). Biological evaluation of myoblast-laden magnetized scaffolds embedded in collagen/Matrigel hydrogels demonstrated good viability, fusion, and differentiation of cells, with adequate axial organization of the resulting myotubes. Constructs composed of PCL/rGNP@-20% were reproduced at the centimeter scale and subjected to cyclic magnetic field loading while immersed in culture medium. At magnetic flux densities of B = 100 mT or higher, the magnetoactive constructs successfully underwent reversible bending. After multiple cycles of magnetic actuation, seeded myoblasts remained in the construct [118]. These outcomes convey the unprecedented ability to perform magneto-mechanical stimulation in a 3D microenvironment model reflective of skeletal muscle. While the authors acknowledge that magnetic stimulation of these constructs facilitates out-of-plane bending, contrasting the in-plane contraction seen in native skeletal muscle, this system expands the possibilities to better understand the mechanical stimulation responsiveness of skeletal muscle and its role in different disease states [118]. Nevertheless, existing research on MEW scaffolds combined with electrical or magnetic stimulation remains primarily focused on short-term effects. Specifically, there is a notable lack of studies evaluating long-term outcomes such as scaffold degradation, structural integrity, and sustained tissue development. Addressing these gaps through longitudinal in vivo studies is essential to fully harness the therapeutic potential of MEW scaffolds under dynamic stimulation and to support their successful clinical translation.

## 9. Challenges and Future Perspectives

**Figure 12:**
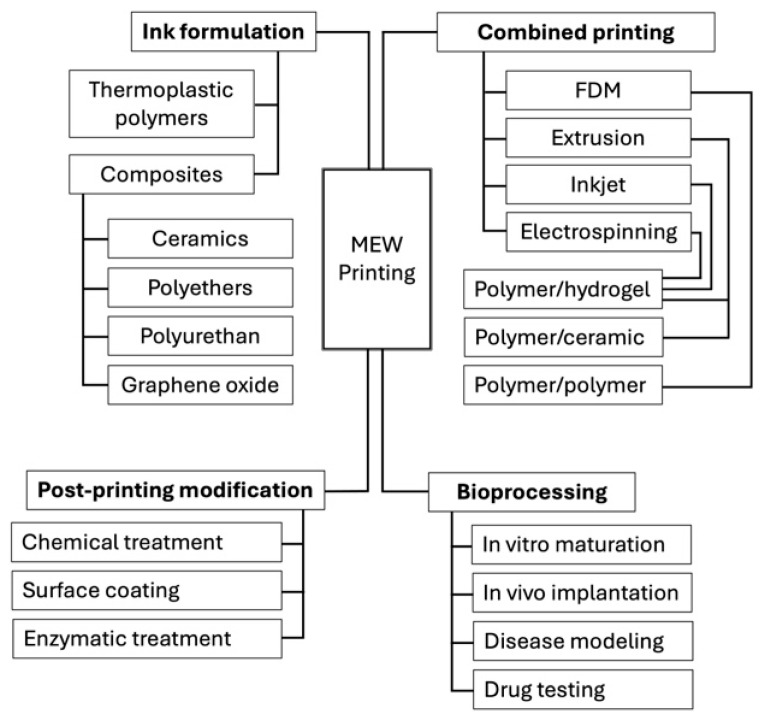
Versality of MEW 3D printing technology and its multiple applications in musculoskeletal tissue engineering.

As shown through the applications described in this review, MEW has begun to establish itself as an innovative and versatile technology that advances the current possibilities of musculoskeletal tissue repair and regeneration (Figure 12). Moreover, MEW has also shown tremendous achievements in the field of tissue regeneration beyond musculoskeletal, with evidence of significant strides in dental regenerative medicine and soft tissue engineering [42,120,121]. Although MEW has made significant progress in tissue engineering and overcoming the limitations of its predecessor AM techniques, much work remains to be done to expand the utilization of MEW in restoring damaged tissues.

For instance, the mechanical properties and stability of MEW scaffolds still require improvement to function within the high-stress, weight-bearing environments of bone, cartilage, ligament, tendon, and muscle. High-precision microfibers fabricated via MEW are suitable for creating an environment that promotes cellular proliferation and differentiation, but can easily undergo deformation and structural alteration, limiting the mechanical support provided by MEW scaffolds. In the same vein, the stability of MEW scaffolds has also been questioned owing to polymer restrictions. While PCL is widely preferred for MEW scaffold fabrication due to its ease of processing at lower temperatures, cost-effectiveness, and availability, its uncoordinated degradation in relation to new tissue formation can compromise the structural integrity of the scaffold, possibly leading to disordered tissue regeneration. Thus, supplemental measures to augment MEW scaffold properties are necessary to overcome this challenge [65,89]. Fortunately, recent studies have experimented with polymer adjuncts and blends to better functionalize scaffolds; therefore, further examination of manipulating MEW polymers with secondary materials may be promising in mimicking mechanical properties and allowing for stable growth of native musculoskeletal tissue [24,90,118].

Incorporating more durable or bioactive materials into the MEW process holds significant promise for enhancing the mechanical stability of scaffolds in high-stress environments. Engineered composite scaffolds by integrating fluorinated calcium phosphate (F/CaP) coatings and collagen into MEW-fabricated PCL structures resulted in improved scaffold’s mechanical properties and enhanced biological functionality. Indeed, the F/CaP coating contributed to increased stiffness and osteoconductivity, while the collagen infusion provided a biomimetic environment conducive to the attachment, proliferation, and osteogenic differentiation of alveolar bone-derived mesenchymal stem cells (aBMSCs), with upregulated expression of bone-forming genes such as runt-related transcription factor 2 (Runx2) and osteocalcin. In vivo, the scaffolds facilitated robust bone formation and vascularization, indicating successful integration with host tissue. However, it is important to consider that modifications to scaffold composition can influence biological functionality. For instance, while the addition of bioactive materials like ions and biomolecules can enhance mechanical properties and cell interactions, they may also increase the degradation rate, which may decrease the mechanical properties of the MEW constructs, and the release of bioactive ions, which in turn can negatively impact the biocompatibility and cellular responses. Therefore, careful optimization of material composition is essential to balance mechanical stability with desired biological outcomes.

Additionally, constraints on manufacturing and scalability of MEW scaffolds proved to be a challenge. Grafts, while not perfect, are the current gold standard for repair of large defects, in large part due to their ability to recover massive tissue damage. Creating large complex scaffolds via MEW that are well fit for sizeable, and irregular defects is difficult, given the size and build volume limitations of MEW printers. Furthermore, attempts to scale up MEW processes for the fabrication of complex designs have resulted in poor precision of fiber placement and reproducibility. In combination with the delicate effect that print parameters have on build outcomes, any changes in the bulk physicochemical properties of the used PCL and its composites can worsen reproducibility and lead to inconsistent scaffold quality, properties that are essential for clinical applications. Hence, as the ability of MEW to regenerate tissue improves, more attention must be paid to enhancing the scalability and consistency of this technique. Overcoming MEW’s limitations in size and scalability will likely require a combination of technological innovations, hybrid manufacturing approaches, and smart design strategies. Such as, the fabrication of smaller MEW modules that can be assembled into larger constructs post-fabrication. This modular approach maintains the microstructural fidelity of MEW while enabling scalability. Also, the development of MEW printers with improved multi-printing head systems could significantly increase printing throughput and build volume while maintaining fine control over fiber placement. These advances are essential to meet the demand for clinically relevant, anatomically sized scaffolds without compromising the precision and biological performance that MEW offers.

One notable limitation of MEW is its incompatibility with direct cell printing. Unlike some bioprinting techniques, such as inkjet or extrusion-based bioprinting that enable simultaneous deposition of cells and biomaterials, MEW operates at high processing temperatures (typically above 80–100 °C), which are not conducive to maintaining cell viability. This thermal constraint restricts the direct integration of living cells during the fabrication process, requiring a sequential approach where cells must be seeded onto prefabricated MEW scaffolds post-printing. While this limitation may reduce the efficiency of creating complex, cell-laden constructs in a single step, it also presents an opportunity for hybrid fabrication strategies. For example, combining MEW with cell-friendly techniques such as inkjet bioprinting or hydrogel infusion allows for spatial control of cell placement within MEW scaffolds. Despite this workaround, the inability to co-print cells directly with MEW remains a significant consideration when designing cell-scaffold constructs for regenerative applications

Finally, as previously mentioned, the primary goal of MEW is the translation to clinical applications in humans, which, in the context of musculoskeletal repair, includes poorly healed nonunion bone defects, volumetric muscle loss, ligament and tendon ruptures, and many other damage and disease states. However, most current in vivo applications in the literature have been conducted using small animal models, such as rats, mice, and rabbits. It must be noted that MEW is still considered a newly emerging 3D printing technique, and many studies are currently focusing on the initial phases of scaffold development and in vitro experiments. These are critical considerations for the clinical translation of MEW-printed scaffolds, particularly in dynamic and mechanically active environments such as muscle tissue. The absence of data on how MEW scaffolds integrate with host tissue and whether they maintain their structural integrity post-implantation highlights a significant gap in the current literature and underscores the need for future studies to address scaffold–tissue interactions and long-term structural performance in vivo. Nevertheless, as the body of evidence in favor of MEW for tissue engineering grows, the focus must necessarily shift towards applying scaffolds to large animal models. As mentioned earlier, only one study attempted this transition using intermediate-size models of Yucatan minipigs to evaluate the impact of surgical fixation methods on the efficacy of MEW-reinforced hydrogel composites and obtained meaningful results [81].

While MEW has shown great promise in fabricating scaffolds with controlled microarchitecture and tailored mechanical properties, a critical challenge for its clinical translation lies in how well these engineered constructs truly replicate the mechanical behavior and cellular responses of native musculoskeletal tissues. Although studies have demonstrated that MEW scaffolds can approximate the compressive modulus of trabecular bone and support osteogenic differentiation [121], guide tenocyte and fibroblast alignment in tendon and ligament models [106], and promote chondrogenic responses in cartilage applications [90], these findings are often limited to in vitro settings or early-stage in vivo experiments. Moreover, while certain designs such as sinusoidal patterns in ligament scaffolds or aligned fibers in muscle-mimicking constructs can emulate specific mechanical characteristics [106,122], a comprehensive match to the dynamic, heterogeneous, and load-bearing environments of native tissues remains an unmet goal. Quantitative comparisons to native tissue properties are still scarce, especially in long-term in vivo models, and most studies lack robust data on scaffold integration, remodeling, and degradation in physiologically relevant environments. For MEW to transition from bench to bedside, future work must focus on systematically evaluating scaffold performance under dynamic mechanical loading, understanding how scaffold architecture influences immune and regenerative responses in vivo, and developing standardized benchmarks that compare MEW constructs to native tissues across multiple functional criteria. As such, confidence in MEW as a promising technology for tissue regeneration has increased, and in vivo applications in musculoskeletal tissues of large animals such as horses and nonhuman primates are worthy of consideration, as they more closely simulate the load-bearing forces experienced by the same tissues in humans. Addressing these gaps is essential for unlocking the full clinical potential of MEW-based tissue engineering strategies.

MEW technology is still in its infancy, and significant gaps remain in understanding how to effectively scale and translate it for clinical use. Key areas such as regulatory approval pathways, long-term in vivo performance, large-scale manufacturing, and cost-effectiveness have yet to be thoroughly addressed in the literature. More systematic studies and interdisciplinary collaborations are needed to evaluate the safety, reproducibility, and economic feasibility of MEW-based therapies. As the field matures, these issues will need to become central to research efforts to ensure successful clinical translation and widespread adoption.

## 10. Conclusions

To date, a multitude of 3D printing and additive manufacturing techniques have been studied in the context of tissue repair and regeneration. This review focused on the recent developments in melt electrowriting, and its ability to fabricate complex scaffolds with incredible precision for regenerative musculoskeletal applications. MEW also displays exceptional versatility in terms of the tailorability of scaffolds and compatibility with other more established printing methods for a hybrid approach towards biomaterials fabrication. Recent advancements have further expanded the potential of MEW by enabling the use of bioactive and composite materials, supporting the design of hierarchical, multi-scale scaffolds that more accurately recapitulate native tissue architecture. Innovations such as stimuli-responsive scaffolds, integration with organ-on-chip platforms, and digital workflows for patient-specific designs reflect MEW’s growing role not only in regenerative medicine but also in disease modeling and personalized therapies. Moreover, efforts to enhance scaffold mechanical stability, control degradation kinetics, and incorporate bioactive release systems are addressing long-standing limitations. Despite these promising developments, challenges remain particularly regarding scalability, long-term mechanical performance, and regulatory considerations, which continue to limit MEW’s full transition into the clinical setting.

## Figures and Tables

**Table 1:** Summary of MEW printing parameters and their effect on printed fibers.

Printing Parameters	Fiber Properties
Melt Temperature	Printability
Printing Speed	Diameter
Collector Distance	Inter-fiber Spacing
Air Pressure	Shape
Applied Voltage	Mechanical Properties

**Table 2:** Examples of commercial MEW printers currently offered on the market and overview of major specifications.

Company	Model Name	Device L × W × H (cm)	XYZ Maximum Build Volume (cm)	Extrusion Technique	XYZ Positional Resolution (µm)	Voltage Max (kV)	Printhead Temperature Control Range (°C)	Other Printhead Options
Axolotl Biosystems	AXO A3	56 × 40 × 43	13 × 9 × 8	Pneumatic	XYZ = 1.25	15	RT—265	Heating Cooling UV
	AXO A6	69 × 40 × 43						
Novaspider	PRO/MEW	70 × 70 × 80	19 × 19 × 21	Pneumatic Piston Screw	Accuracy: XY = 12.5, Z = 3.125	30	Pneumatic & Piston Heads: RT—260 Screw Head: RT—300	Syringe pump
Nanofiber Labs	MBP-001	85 × 85 × 180	N/A	Pneumatic	Accuracy: XY ≤ 1 Z = 5	50	RT—300	Injection pump Extrusion nozzle
	M01-005	N/A	20 × 20 × 10		Accuracy: XY ≤ 20 Z = 20	30		
	M08-001	N/A	N/A		Accuracy: XY ≤ 2 Z = 5	30		
Tongli	TL-Trinity/ TL-03	90.8 × 72.4 × 161.3	18 × 18 × ~1	Pneumatic, Spinneret	XYZ = 0.1, Repeatability: 3 × 3 × 8	50	RT—400	UV curing
RegenHU	R-GEN 100	77 × 72 × 76	13 × 9 × 6.5	Pneumatic	XYZ ≤ 1	25	RT—250	Drop dispenser Strand dispenser Electrospinning Volumetric strand dispenser
	R-GEN 200	136.6 × 96.3 × 220.2						
GeSiM	BS3.3 BS3.3 Prime	7.4 × 5.7 × 6.6	Tray size: 3.5 × 2.5 cm^2^	Pneumatic Piston Gradient Mixers Liquid Dispensing	XY = 1, Repetitive Accuracy: ±10	30	RT—200	Heating Cooling UV
	BS5.3	8.78 × 7.15 × 7.64	Tray size: 2.4 × 4.12 cm^2^					
	BS5.3/E	11.36 × 7.15 × 7.64						

Note: This list is not a comprehensive of all MEW printers on the market. Data was obtained from company websites or by contacting the company directly. This table includes data available up to 9 December 2024. N/A = data not available or unable to be found. Specifications for some printers may be customized per consumer request. Thus, not all data provided is absolute.

**Table 3:** A summary table outlining the effects of MEW scaffold-related factors such as architectural design, supplementation with other 3D printing techniques, and biomimetic bone environments on bone and cartilage regeneration.

Target	Scaffold Composition	MEW Design Features	Supplementation/Bioprinting	Key Outcomes	Ref.
Bone	PCL	MEW + “brick-and-mortar” structure		Enhanced osteogenesis, vascularized bone tissue formation	[65]
	PCL with BMP-2	Box-pore architecture		Sustained BMP-2 delivery, enhanced bone regeneration	[66]
	PCL with calcium phosphate cement (CPC)	Multilayer MEW scaffold	Extrusion bioprinting	Enhanced mineralization and mechanical strength	[67]
	PCL + nanoHA	Layered MEW mesh	Inkjet bioprinting of osteogenic factors	Improved bone-like matrix deposition and bone repair	[68]
	PCL with F/CaP coating and collagen	Standard MEW mesh		Upregulated osteogenic markers (Runx2, OCN), enhanced stiffness, robust bone formation, but potential degradation trade-offs	[90]
Osteochondral	PCL	Bilayer MEW scaffold	Extrusion bioprinting (bioceramic + hydrogel)	Increased interface strength 6-fold; promoted osteogenesis and chondrogenesis	[86]
	PCL + gelMA	Layered MEW scaffold (0°-45°-90°-135°)	Bioprinting with ceramic and hydrogel	Enhanced compressive/shear moduli, biomimetic architecture	[87]
	PCL (MEW, electrospinning, porogen leaching)	Multizonal scaffold		Supported chondrocyte growth, osteointegration after 6 months	[88]
	PCEC (PCL + PEG)	Trilayer MEW scaffold	MSC-laden hydrogels + zone-specific growth factors	Site-specific collagen II/I expression, excellent integration, surface lubrication	[89]
Cartilage	PCL/PCL-HA	MEW + Inkjet-printed microspheres	TGF-β1, BMP-7, IGF-1	Smooth cartilage formation, enhanced integration	[77]
	Gelatin/PLGA + TGF-β1 + HA	MEW-reinforced hydrogel		Coverage of injured area with normal morphology	[78]
	NorHA hydrogel + PCL MEW mesh	Press-fitted cylindrical design		50× increase in modulus, strong integration, GAG and collagen II expression	[79]
	PCL microfiber + chondrocyte hydrogel	Bilayer MEW hydrogel scaffold		New cartilage formed without growth factors, high viability	[80]
	NorHA + PCL MEW mesh	Tested with different fixation methods	Bioresorbable pins, fibrin glue	Heterogeneous filling; fibrin glue favored integration	[81]
	PCL MEW scaffold with Inkjet MSCs	Box-like MEW mesh		Self-assembled spheroids, hyaline-like tissue, 3-layer zonal structure	[83]
	PCL MEW + ACPC-laden gelMA	Printed onto curved femoral surfaces		Maintained shape, new cartilage formation, improved mechanics	[84]

## Data Availability

No new data were created or analyzed in this study. Data sharing is not applicable to this article.

## Abbreviations

Additive manufacturing	(AM)
Adipose tissue-derived mesenchymal stem cells	(AT-MSC)
Alizarin red staining	(ARS)
Alkaline phosphatase	(ALP)
Alveolar bone-derived mesenchymal stem cells	(aBMSCs)
Anterior cruciate ligament	(ACL)
Articular cartilage	(AC)
Articular cartilage-resident chondroprogenitor cells	(ACPC)
Beta-tricalcium phosphate	(β-TCP)
Bone morphogenic protein-2	(BMP-2)
Bone morphogenic protein-7	(BMP-7)
Calcium phosphate	(CaP)
Calcium phosphate cement	(CPC)
Cell counting kit-8	(CCK-8)
Chondroitinase ABC	(cABC)
Collagen meniscus implant	(CMI)
Critical translation speed	(CTS)
Deoxyribonucleic acid	(DNA)
Extracellular matrix	(ECM)
Fibrin/alginate	(FA)
Fluorinated calcium phosphate	(F/CaP)
Fused deposition modeling	(FDM)
Gelatin methacrylate	(gelMA)
Glycosaminoglycans	(GAGs)
Hematopoietic stem cells	(HSCs)
Hematoxylin and eosin	(H&E)
Human bone marrow stem cells	(BMSCs)
Human osteoblasts	(hOBs)
Human osteoblast-derived mineralized microtissue	(hOBMT)
Human umbilical vein endothelial cells	(HUVECs)
Hydroxyapatite	(HA)
Insulin-like growth factor 1	(IGF-1)
Magnesium phosphate	(mgp)
Magnetized graphene nanoplatelets	(rGNP@)
Melt electrowriting	(MEW)
Meniscus progenitor cells	(MPCs)
Meniscus progenitor cells inner zones	(iMPCs)
Meniscus progenitor cells outer zones	(oMPCs)
Mesenchymal stem cells	(MSCs)
Methacrylated alginate	(AA-MA)
Methacrylate hyaluronic acid	(HA-MA)
Micro-computed tomography	(Micro-CT)
Nano-needle hydroxyapatite	(nnHA)
PCL-poly (ethylene glycol)	(PCEC)
Poly(ε-caprolactone)	(PCL)
Poly(ε-caprolactone-co-acryloyl carbonate)	(PCLAC)
Poly(ethylene glycol)	(PEG)
Poly(L-lactic acid)	(PLLA)
Poly(lactic acid)	(PLA)
Polyurethane	(PU)
Prostate cell line	(PCa)
Runt-related transcription factor 2	(Runx2)
Scanning electron microscopy	(SEM)
Tendon stem cells	(TCs)
Three-dimensional	(3D)
Transforming growth factor beta 1	(TGF-β1)
Two-dimensional (2D)	(2D)
Ultraviolet	(UV)
Volts	(V)
Zinc oxide	(ZnO)
Zinc oxide nanoflakes	(ZF)
Zinc oxide nanoparticles	(ZP)
